# Campylobacter jejuni Triggers Signaling through Host Cell Focal Adhesions To Inhibit Cell Motility

**DOI:** 10.1128/mBio.01494-21

**Published:** 2021-08-24

**Authors:** Courtney M. Klappenbach, Nicholas M. Negretti, Jesse Aaron, Teng-Leong Chew, Michael E. Konkel

**Affiliations:** a School of Molecular Biosciences, College of Veterinary Medicine, Washington State University, Pullman, Washington, USA; b Advanced Imaging Center, Janelia Research Campus, Howard Hughes Medical Institute, Ashburn, Virginia, USA; Iowa State University

**Keywords:** pathogenesis, focal adhesion modulation, focal adhesion turnover, cell migration, iPALM, bacterium-host cell interactions

## Abstract

Campylobacter jejuni is a major foodborne pathogen that exploits the focal adhesions of intestinal cells to promote invasion and cause severe gastritis. Focal adhesions are multiprotein complexes involved in bidirectional signaling between the actin cytoskeleton and the extracellular matrix. We investigated the dynamics of focal adhesion structure and function in C. jejuni-infected cells using a comprehensive set of approaches, including confocal microscopy of live and fixed cells, immunoblotting, and superresolution interferometric photoactivated localization microscopy (iPALM). We found that C. jejuni infection of epithelial cells results in increased focal adhesion size and altered topology. These changes resulted in a persistent modulatory effect on the host cell focal adhesion, evidenced by an increase in cell adhesion strength, a decrease in individual cell motility, and a reduction in collective cell migration. We discovered that C. jejuni infection causes an increase in phosphorylation of paxillin and an alteration of paxillin turnover at the focal adhesion, which together represent a potential mechanistic basis for altered cell motility. Finally, we observed that infection of epithelial cells with the C. jejuni wild-type strain in the presence of a protein synthesis inhibitor, a C. jejuni CadF and FlpA fibronectin-binding protein mutant, or a C. jejuni flagellar export mutant blunts paxillin phosphorylation and partially reestablishes individual host cell motility and collective cell migration. These findings provide a potential mechanism for the restricted intestinal repair observed in C. jejuni-infected animals and raise the possibility that bacteria targeting extracellular matrix components can alter cell behavior after binding and internalization by manipulating focal adhesions.

## INTRODUCTION

Significant research effort has focused on understanding how infectious agents cause disease in the host. Progress in understanding the fundamental mechanisms of bacterial pathogens has revealed common virulence strategies. Some of the most common targets for cellular-level manipulation are the cytoskeleton and signaling pathways (1–3), which often enable bacterial pathogens to invade host cells (4). Promoting bacterial internalization can protect pathogens from the immune system and prolong infection (5). Many extensively studied pathogens, such as Salmonella, Shigella, Vibrio, and Listeria, have been found to use bacterial adhesins and secreted proteins (effectors) to alter signaling pathways, resulting in cytoskeleton rearrangements that promote their internalization (1, 2, 5). There are several major protein complexes connecting a cell to its external environment that can be targeted by bacteria for signaling manipulation.

In polarized epithelial cells, such as those in the intestine, there are four types of intracellular junctions, namely, tight junctions, adherens junctions, hemidesmosomes, and focal adhesions. Tight and adherens junctions connect cells, whereas hemidesmosomes and focal adhesions facilitate cell-matrix connections (6, 7). Focal adhesions are unique from the others in that they connect the actin cytoskeleton to the extracellular matrix (ECM), making them dynamic and complex structures. The primary functions of focal adhesions are signaling, coordinating motility/migration, and adherence to the basal membrane. Signaling constitutes a major role for focal adhesions, as they can transmit bidirectional signals across the cell membrane (8). A vast range of information is transmitted through focal adhesion signaling, including signals for survival, proliferation, differentiation, platelet aggregation, embryo development, and sensing of extracellular and intracellular tension (7, 9–11). A major function of these signals is to direct migration of epithelial cells in the intestinal villi, an important process for homeostasis (12). In the intestine, new cells are produced from pluripotent stem cells in the villus crypt, and these migrate along the villus-crypt axis toward the villus tip, where they are eventually extruded (12, 13). The majority of studies on the cellular migration process have focused on collective epithelial cell migration in response to villus damage (14–18). When the villi become damaged, the basal lamina may become exposed or denuded (14). To repair this, healthy epithelial cells surrounding the wound will depolarize and migrate to cover the basal membrane (14–18). Cell migration is dependent on focal adhesion dynamics (19), whereby cells must simultaneously assemble new focal adhesions at the leading edge of the cell and disassemble old focal adhesions from the trailing edge of the cell (20). The balanced process of these actions allows cells to have directed migration (20). In addition to motility and migration, focal adhesions physically adhere cells to their external environment by attaching to the ECM. Focal adhesions depend on many proteins to manage these roles and functions.

Focal adhesions are composed of an organized array of proteins, collectively known as the focal adhesion “adhesome” (21). Integrins, transmembrane proteins that bind both ECM components and intracellular signal transduction proteins, comprise the base of focal adhesions (8). Structural proteins, such as talin and vinculin, connect integrins to the actin cytoskeleton and are required to strengthen focal adhesions (22). Last, signaling proteins allow the focal adhesion to send bidirectional signals through the cell membrane. These proteins include adaptors, scaffolds, kinases, and proteases (10). The coordinated action of adhesome proteins allows the cell to transmit signals between the extracellular environment and the intracellular actin cytoskeleton, as well as to perform important cell functions such as motility/migration and adhesion.

Since many pathogens rely on the actin cytoskeleton and signaling proteins to cause infection in a host, focal adhesions are prime targets. Campylobacter jejuni is a major foodborne pathogen most commonly acquired from ingestion of undercooked chicken or foods cross-contaminated with raw poultry. Despite being the most common culture-proven cause of bacterial gastroenteritis worldwide (23), our knowledge of its specific virulence mechanisms and host-cell interactions is incomplete. It is known that severe C. jejuni infection results in diarrhea with blood in the stool and is accompanied by villus blunting (24–26). It is expected that villus restitution and collective cell migration would repair the tissue damage; however, it is not known if the phenotype observed in C. jejuni infection is due to slowing this process of villus repair. The purpose of this study was to determine if C. jejuni reduces cell motility and, if it does, to determine the cellular mechanism that drives the alteration in cell behavior. We discovered that C. jejuni manipulates the dynamics of the focal adhesion, in part, via the CadF and FlpA fibronectin-binding proteins and secreted effector proteins. These findings constitute a new C. jejuni-driven cellular phenotype.

## RESULTS

### C. jejuni reduces host cell motility.

**FIG 1:**
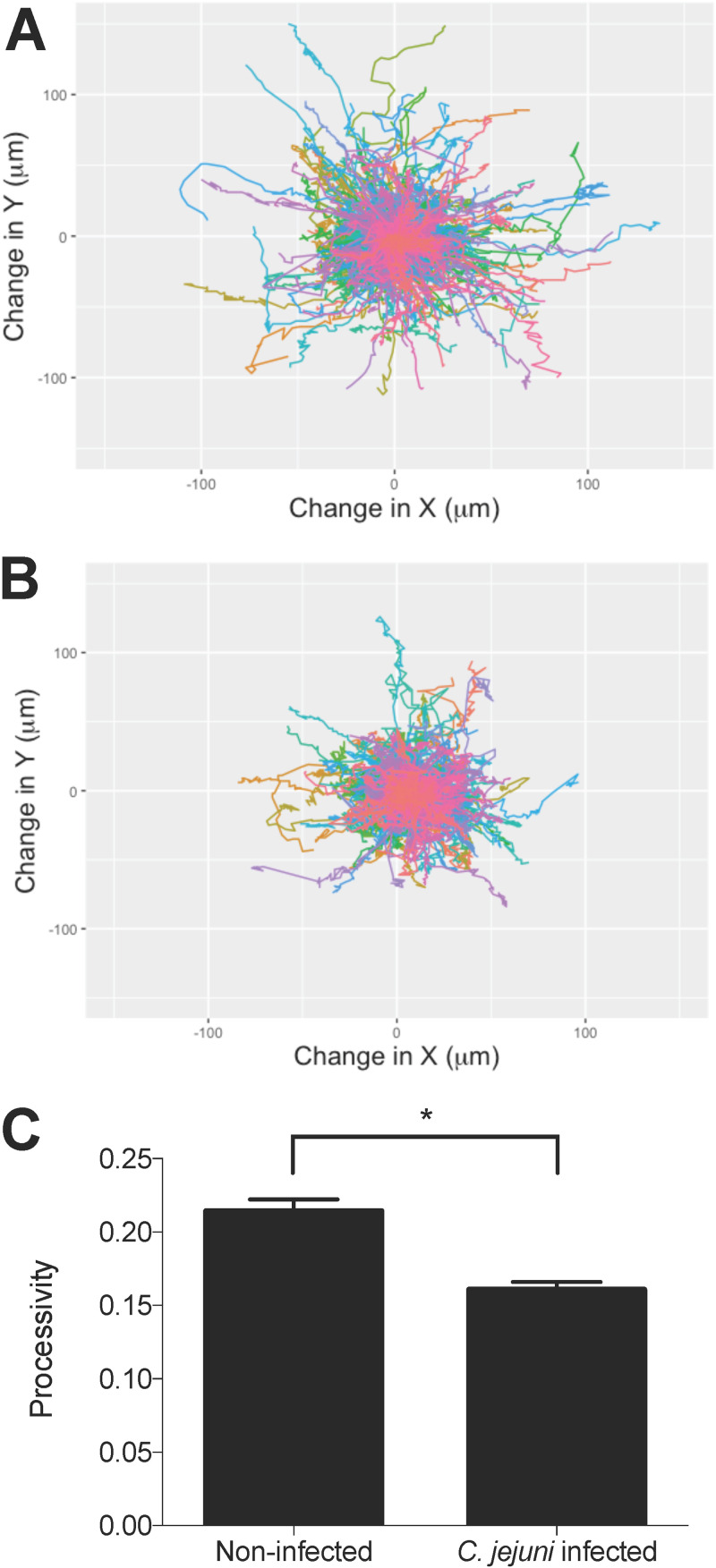
C. jejuni infection of A549 epithelial cells decreases their motility. A549 cells were grown on a laminin-based extracellular matrix prior to infection with C. jejuni. An image was taken every 5 min for 5 h, and images were analyzed to track cell movement. Wind rose plots (A and B) show the tracked paths of individual cells (different colors) over 5 h. (A) Wind rose plot of noninfected cells shows that most cells move far from the starting position (center of the plot: 0,0). (B) Wind rose plot of C. jejuni infected cells shows that they do not move as far from their starting position compared to noninfected cells. (C) C. jejuni causes a significant decrease in processivity, calculated as path distance divided by displacement averaged for all cells. Error bars represent standard error of the mean (SEM). *, *P < *0.0001 (Student’s *t* test). More than 500 cells were imaged per condition.

To understand how C. jejuni manipulates the behavior of host cells during infection, we first examined the effect of C. jejuni on individual host cell motility using the A549 epithelial cell line. This cell line was chosen for the initial assays because the cells are highly motile (27). Cells were infected with C. jejuni, imaged for 5 h, and then individual cells were tracked to quantify their motility. Cell motility was significantly decreased by infection with C. jejuni (Fig. 1). Observation of the paths of individual cells over the course of the 5 h infection shows that C. jejuni-infected cells traveled a shorter distance (path distance) and remained closer to their starting position (path displacement) (Fig. 1A and B). Processivity (path distance divided by path displacement) was calculated to quantify this observation. This measurement indicates a cell’s directional movement and is commonly used to assess cell motility (28). C. jejuni-infected cells showed a significant decrease in processivity compared to that of noninfected cells (Fig. 1C).

We also investigated the effect of Salmonella enterica subsp. *enterica* serovar Typhimurium and Staphylococcus aureus on cell motility. S. aureus is a nonmotile bacterium that causes sickness by staphylococcal enterotoxins (SE), triggering vomiting and inflammation of the intestine (29). *S.* Typhimurium, however, is a motile bacterium and causes disease by invading host cell intestinal tissues. Both C. jejuni and Salmonella infection require intimate bacterial-host cell contact for disease, while S. aureus infection does not. We investigated the effect of both *S.* Typhimurium and S. aureus on single-cell motility. A549 epithelial cells were infected with the bacterium for 1 h, and then the cells were rinsed and imaged for 5 h. Chloramphenicol was used at a bacteriostatic concentration to ensure that the bacterial CFU was consistent throughout the experiment. Cells infected with *S.* Typhimurium had significantly lower motility than that of noninfected cells; however, there was no significant difference in cell motility for cells infected with S. aureus (see Fig. S1 in the supplemental material). Our findings are consistent with the fact that *S.* Typhimurium binds to fibronectin and induces the formation of focal adhesion-like complexes during cell invasion (30, 31). This result suggests that the inhibition of host cell motility is conserved among some bacterial species.

10.1128/mBio.01494-21.1 FIG S1 Salmonella enterica subsp. *enterica* serovar Typhimurium (*S.* Typhimurium), but not Staphylococcus aureus, decreases single-cell motility. A549 cells were infected with *S.* Typhimurium or S. aureus for 60 minutes, rinsed with phosphate-buffered saline (PBS), and imaged for 5 hours, with one image taken every 5 minutes. Cells were tracked over time, and processivity (path distance divided by path displacement) was calculated to quantify motility. Error bars represent standard error of the mean (SEM). *, *P < *0.0001 (Student’s *t* test). More than 500 cells were imaged per condition. Download FIG S1, TIF file, 0.01 MB. Copyright © 2021 Klappenbach et al. 2021 Klappenbach et al. https://creativecommons.org/licenses/by/4.0/ This content is distributed under the terms of the Creative Commons Attribution 4.0 International license.

### C. jejuni increases the size of the focal adhesion in a flagellum-dependent manner.

**FIG 2:**
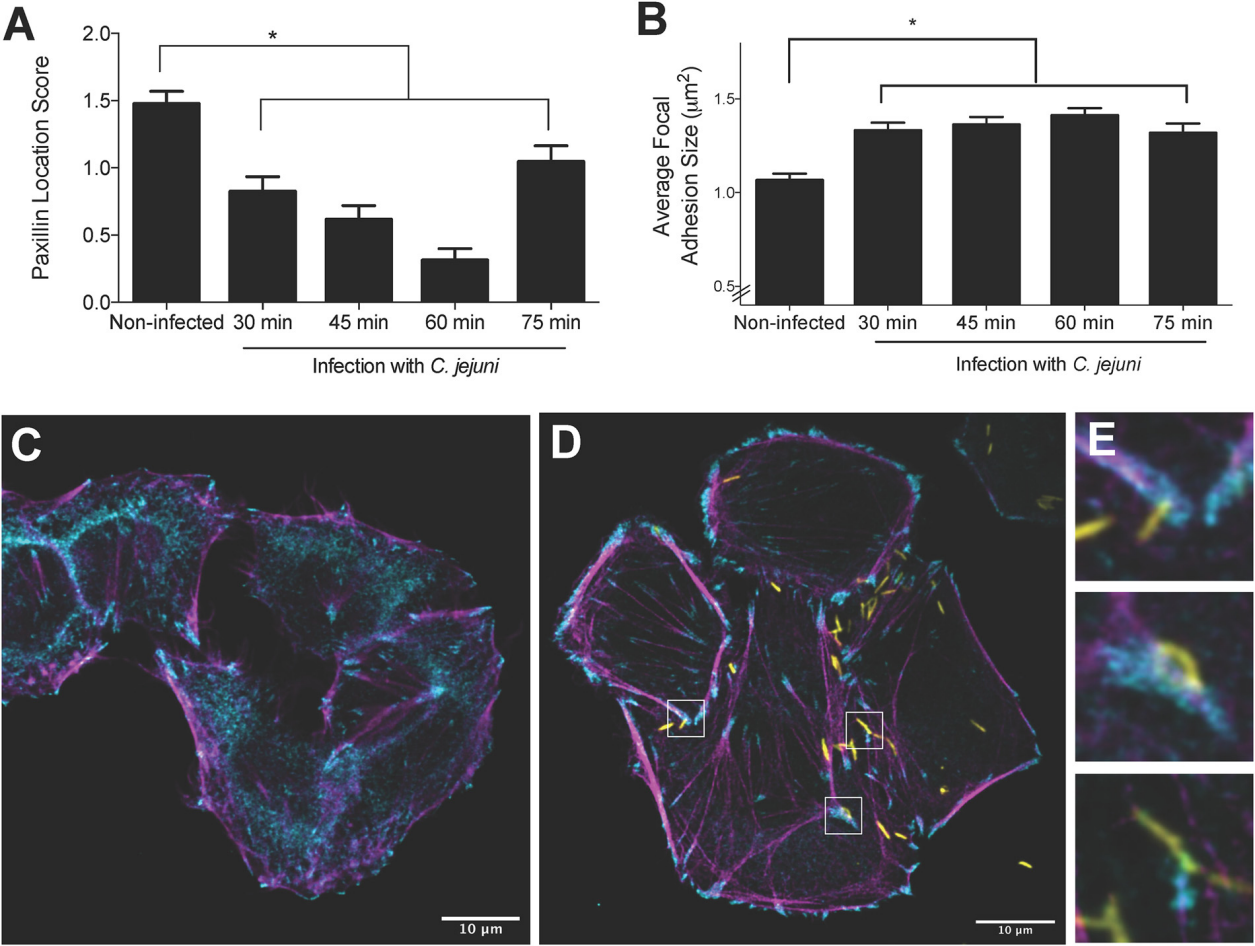
C. jejuni associates with and increases the size of the focal adhesion footprint and changes the localization of paxillin. INT 407 cells were infected with C. jejuni for 30 to 75 min prior to fixing and immunostaining for paxillin, actin, and C. jejuni. (A) Paxillin is localized to the cytosol in noninfected cells and localized at the focal adhesion in C. jejuni-infected cells. Paxillin localization was determined by an individual who was blind to the samples, where 0 = complete focal adhesion localization and 2 = cytosolic localization. Error bars represent SEM of > 46 cells. *, *P < *0.05 compared to the noninfected sample (one-way analysis of variance [ANOVA] and Dunnett’s multiple-comparison test). (B) C. jejuni causes a significant increase in focal adhesion size at 30, 45, 60, and 75 min postinfection. Error bars represent standard deviation of > 1,000 focal adhesions. *, *P < *0.05 compared to the noninfected sample (one-way ANOVA with Tukey’s multiple-comparison test). (C) Representative image of noninfected cells. (D) Representative images of C. jejuni-infected cells. (E) Magnification of insets from panel D showing that C. jejuni is associated with paxillin. Paxillin is shown in cyan, actin in magenta, and C. jejuni in yellow. The focal adhesions in infected cells appear larger than those in noninfected cells, and there is less paxillin in the cytosol of C. jejuni-infected cells.

Since epithelial cell motility is driven by a balance between focal adhesion assembly and disassembly (20), we investigated if C. jejuni alters host cell focal adhesion structure. The human INT 407 cell line was used for these assays, as this particular cell line has been used extensively by researchers to dissect C. jejuni-host cell interactions (32–34). Cells were infected with C. jejuni and then fixed and stained for the host cell protein paxillin, which is a major signaling and adaptor protein in focal adhesions (35). Confocal microscopy showed significant changes in paxillin localization between infected and noninfected cells (Fig. 2A). In noninfected cells, paxillin was largely localized in the cytosol, whereas in C. jejuni-infected cells, it was concentrated at focal adhesions. This change was found to be significantly different when scored by an individual blind to the sample identity, where a score of 0 represented complete cytosolic localization and a score of 2 represented no cytosolic localization. The individual determined the score for each cell in the randomized, blind images using a scoring key. Consistent with this observation, quantitation of focal adhesion size in infected and noninfected cells showed that C. jejuni caused a significant increase in focal adhesion size from 30 to 75 min postinfection (Fig. 2B). Many C. jejuni also appeared to localize near focal adhesions (Fig. 2D and E). This is consistent with previously published results (36) showing that C. jejuni colocalized with paxillin and vinculin, another major focal adhesion protein. This led us to investigate the bacterial factors required for the observed changes.

**FIG 3:**
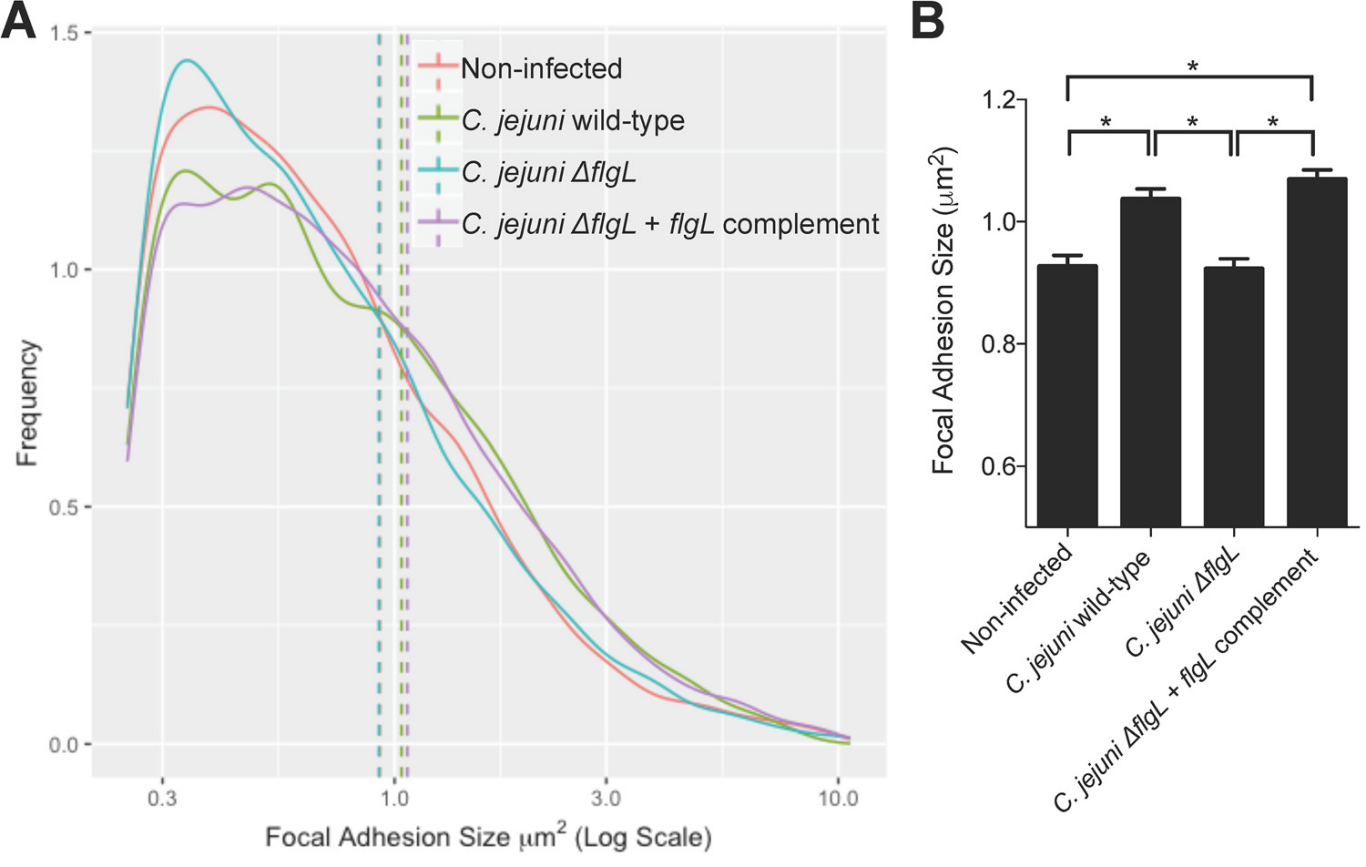
C. jejuni-driven focal adhesion size increase is dependent on a functional flagellum. INT 407 cells were infected with a C. jejuni wild-type strain, a C. jejuni Δ*flgL* mutant, or a C. jejuni Δ*flgL + flgL*-complemented isolate for 60 min prior to fixing and immunostaining for paxillin, actin, and C. jejuni. Focal adhesion size was determined by measuring the paxillin footprint. (A) Frequency distribution of focal adhesion sizes for the four conditions is shown. Solid lines show frequency distribution, and dashed lines show the mean of all focal adhesions within a category. Noninfected and C. jejuni Δ*flgL* mutant-infected cells have similar distributions of focal adhesion sizes, while cells infected with the C. jejuni wild-type strain and the C. jejuni Δ*flgL + flgL*-complemented isolate have similar distributions. (B) The bar graph shows the mean focal adhesion size for each condition. The C. jejuni wild-type strain increases the average focal adhesion size when the *flgL* gene is present, indicating that motility and/or protein secretion are required. Error bars represent SEM. *, *P < *0.0001 (one-way ANOVA with Tukey’s multiple-comparison test; >3,000 focal adhesions measured per condition).

The flagellum is an important virulence factor for C. jejuni; it confers motility and also serves as the bacterium’s type III secretion system to deliver effector proteins into a host epithelial cell (37–41). To determine if the changes in focal adhesion structure were driven by bacterial factors, a C. jejuni Δ*flgL* mutant was tested alongside wild-type bacteria. The *flgL* gene encodes the flagellar hook junction protein, and a C. jejuni Δ*flgL* mutant is nonmotile and incapable of protein export, including secretion of the effectors (32, 42). In contrast to the C. jejuni wild-type strain, the Δ*flgL* mutant had no effect on focal adhesion size, showing a similar focal adhesion distribution to that of noninfected cells (Fig. 3). The C. jejuni *flgL*-complemented isolate, however, restored the phenotype observed in the wild-type bacteria. This result demonstrates that a functional flagellum (i.e., secretion apparatus) is required to increase the size of the focal adhesion.

To further explore how C. jejuni manipulates the focal adhesion, we tested single-cell motility using a Δ*cheB* mutant and an Δ*flhF* mutant that had been generated previously (32). The C. jejuni Δ*flgL* mutant was included as a control. The C. jejuni CheB protein has methylesterase activity, and CheR has methyltransferase activity. Both are involved in chemotaxis (43). Deletion of the *cheB* gene has a polar effect on *cheR* expression; therefore, we will refer to this isolate as a *cheBR* mutant. It is notable that the *cheBR* mutant has a functional flagellum (i.e., secretion apparatus, as evident by the presence of the flagellum), is motile, and does not exhibit a defect in adherence or invasion compared to those of a wild-type strain (32). The C. jejuni FlhF protein binds to the promoters of flagellar genes and regulates their transcription. Disruption of the *flhF* gene in C. jejuni alters the expression of flagellar genes and the process of flagellar biosynthesis, resulting in a nonflagellar phenotype, absence of motility, and decreased epithelial cell adherence and invasion (32). The *cheBR* mutant was able to limit A549 host cell motility to a level similar to that seen in infection of cells with the wild-type bacterium (Fig. S2). In contrast, A549 cells infected with the Δ*flhF* mutant or Δ*flgL* mutant were more motile than cells infected with the wild-type isolate (Fig. S2). Although a subset of flagellar proteins needed for motility are necessary for a functional export apparatus (42), these data suggest that bacterial motility and/or a functional flagellar export apparatus is required to alter (reduce) host cell motility.

10.1128/mBio.01494-21.2 FIG S2 Campylobacter jejuni requires a motile flagellum and invasive ability to limit A549 individual cell motility. A549 cells were infected with the C. jejuni wild-type strain and the C. jejuni Δ*cheB*, Δ*flhF*, and Δ*flgL* flagellar mutants. The C. jejuni Δ*cheB* mutant, which lacks expression of *cheB* and *cheR*, has reduced motility and chemotaxis but normal invasion and effector secretion. This mutant resulted in host cell motility equal to that of the wild-type strain. Mutants that are completely nonmotile and noninvasive (Δ*flhF and* Δ*flgL*) showed the most restoration of motility. By one-way analysis of variance (ANOVA) with Tukey’s multiple-comparison test: #, significant difference from all other conditions (*P < *0.001); †, significant difference from noninfected, Δ*flhF*, and Δ*flgL* conditions (*P < *0.001); ‡, significant difference from noninfected, wild-type, and Δ*cheB* conditions (*P < *0.001). Error bars represent SEM of >300 cells. Download FIG S2, TIF file, 0.09 MB. Copyright © 2021 Klappenbach et al. 2021 Klappenbach et al. https://creativecommons.org/licenses/by/4.0/ This content is distributed under the terms of the Creative Commons Attribution 4.0 International license.

### C. jejuni slows paxillin turnover and changes the topology of the focal adhesion.

**FIG 4:**
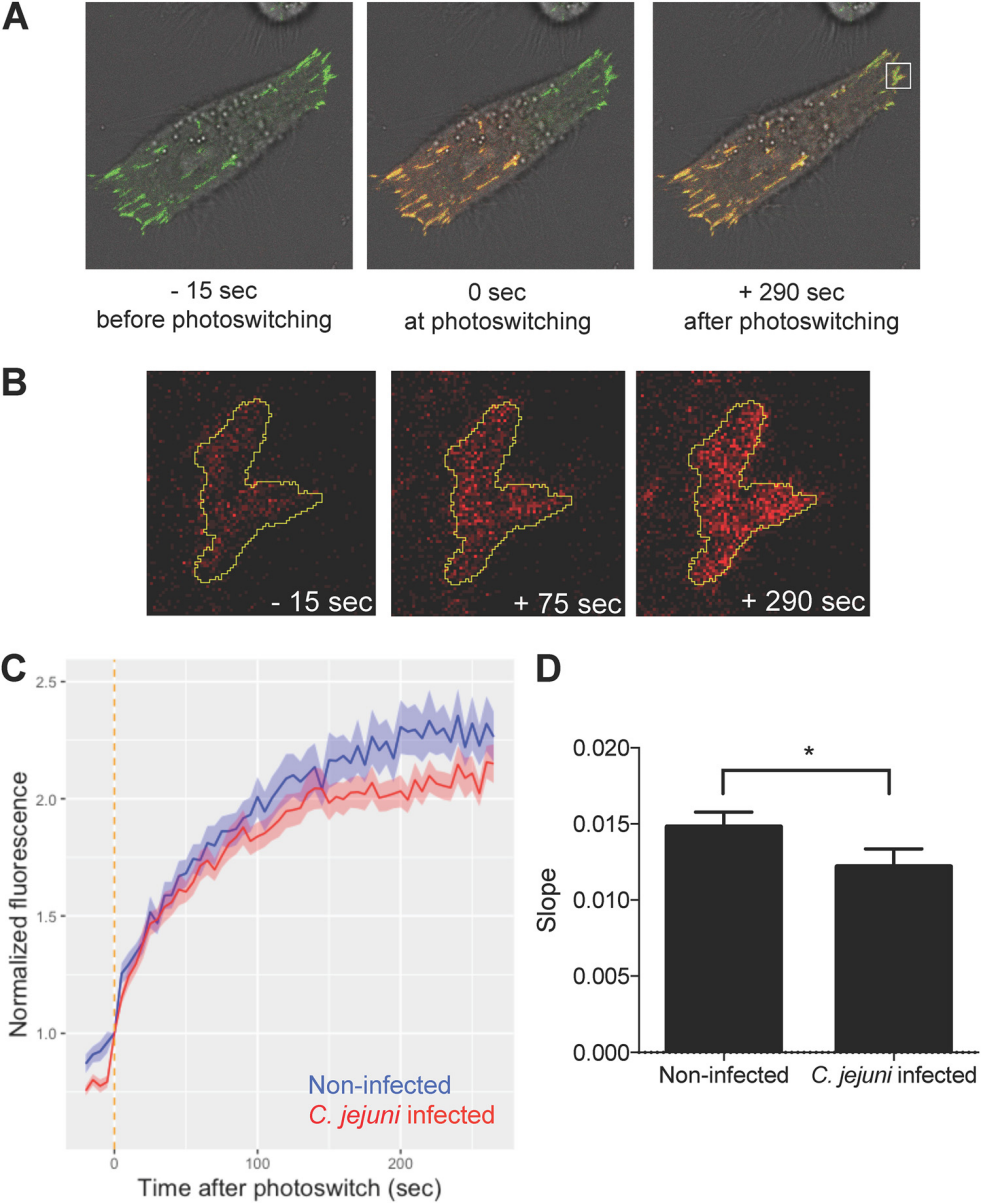
C. jejuni infection slows the turnover of paxillin at the focal adhesion. INT 407 cells were transfected with a tdEos-paxillin plasmid, which photoswitches from green to red when exposed to UV light. Cells were infected for approximately 60 min with C. jejuni prior to imaging. (A) Half of the cell was exposed to UV light to switch the tdEos-paxillin from green to red, and then the cell was imaged every 5 s for approximately 300 s. As the focal adhesion turns over, the green paxillin in the nonphotoswitched half of the cell is replaced with red paxillin from the photoswitched half of the cell. (B) Enlarged view of a focal adhesion in the nonphotoswitched half of the cell showing the red fluorescence channel only increasing over time. (C) Normalized fluorescence was calculated as the average intensity of red fluorescence at focal adhesions in the nonphotoswitched half of the cell divided by each focal adhesion’s initial red fluorescence intensity. Lines represent the average of all focal adhesions for a condition. The focal adhesions of C. jejuni-infected cells took longer to show increased red fluorescence and reached an overall lower red fluorescence intensity. The vertical orange line represents the time of photoswitching. (D) The average slope of red fluorescence in the nonphotoswitched half of the cell was calculated for all focal adhesions by linear regression between normalized fluorescence and time. C. jejuni-infected cells had decreased slope compared to that of noninfected cells, indicating a lower rate of paxillin turnover. Error bars represent SEM. *, *P < *0.0001 (Student’s *t* test). More than 200 focal adhesions were measured per condition.

To determine the mechanism of the focal adhesion size increase, we investigated the turnover rate of paxillin at the focal adhesion in INT 407 cells. Proteins at the focal adhesion regularly turn over and have unique residency times (21, 44). INT 407 cells were transfected with a plasmid containing tdEos conjugated to paxillin. The tdEos protein photoswitches from green to red when exposed to UV light. By photoswitching half of a cell’s focal adhesions, it is possible to measure focal adhesion turnover by observing the rate at which the photoswitched protein replaces the nonphotoswitched protein over time (Fig. 4A). At the nonphotoswitched half of the cell, viewing the red channel only showed that red photoswitched paxillin increased over time at focal adhesions; this observation demonstrates that the photoswitched paxillin (red) migrated to the site of the nonphotoswitched paxillin (green) (Fig. 4B). Moreover, there was a significant decrease in the average rate of red fluorescence appearing in the nonphotoswitched half of the cell for the C. jejuni-infected cells compared to that in noninfected cells. This implies lower focal adhesion turnover and a decrease in the dynamicity of the focal adhesions. As a result of C. jejuni manipulating focal adhesion structure and signaling, the focal adhesion’s ability to turn over paxillin is slowed.

**FIG 5:**
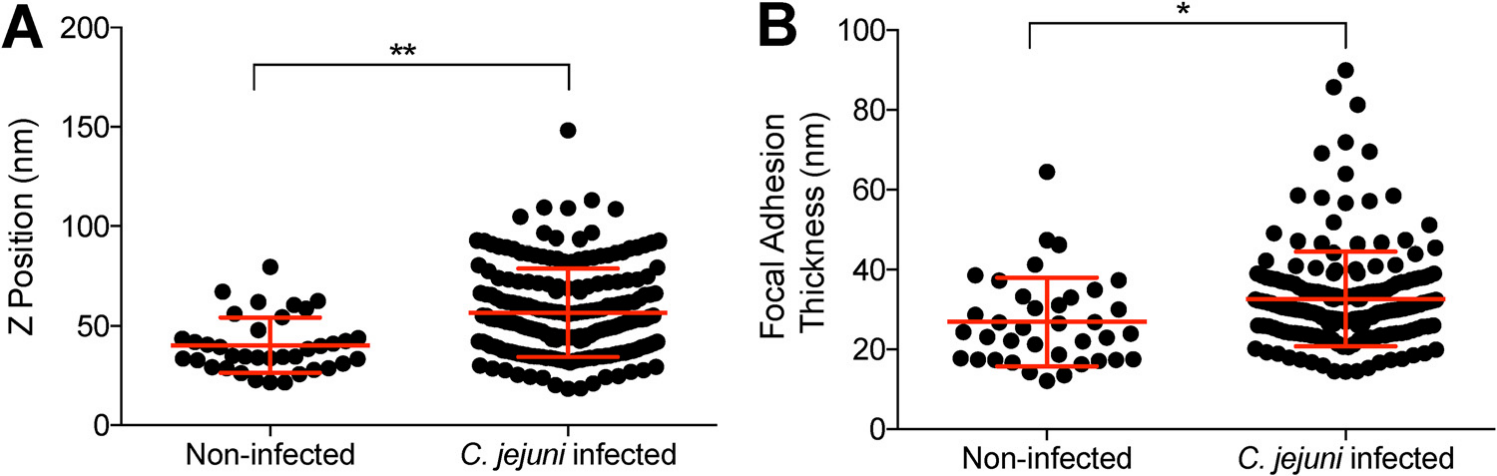
C. jejuni changes the nanoscale topology of the focal adhesion plaque. Superresolution iPALM microscopy was performed to determine the nanoscale architecture of the focal adhesion in C. jejuni-infected cells compared to that in noninfected cells. (A) The *Z* position of a paxillin plaque was determined as the average of the *Z* positions of all fluorophores detected within a focal adhesion. The average *Z* position of C. jejuni-infected focal adhesions was significantly higher than that in noninfected cells. (B) The average thickness of the focal adhesion was determined as the interquartile range of all fluorophores within the focal adhesion. The average thickness of C. jejuni-infected focal adhesions was significantly higher than that in noninfected cells. Error bars represent standard deviation (SD). *, *P < *0.01; **, *P < *0.0001 (Student’s *t* test).

While we observed that C. jejuni infection of epithelial cells caused an increase in focal adhesion size, it was not clear if the structural topology within the focal adhesion was changing. To observe the microscale structure of the focal adhesions in C. jejuni-infected cells, superresolution interferometric photoactivated localization microscopy (iPALM) was utilized to observe the organization of proteins within the focal adhesion at sub-20-nm resolution (45). tdEos-paxillin was used as our indicator of the focal adhesion structure due to its critical role in focal adhesion organization and C. jejuni invasion. We observed that the spatial localization of paxillin was significantly different in C. jejuni-infected cells compared to that in noninfected cells (Fig. 5; see also Fig. S3). The average *Z* position of paxillin, measured as the distance from the gold fiducial beads embedded in the coverslip, was 40.3 nm ± 2.2 nm in noninfected cells. This is consistent with the published value of 36.0 nm ± 4.7 nm (46). The position of paxillin in C. jejuni-infected cells was significantly higher, at 56.7 nm ± 1.6 nm (Fig. 5A). In addition, we calculated the thickness of the paxillin plaque. The plaque in noninfected cells had an average thickness of 26.9 nm ± 1.8 nm, while C. jejuni-infected cells had significantly thicker focal adhesions of 32.6 nm ± 0.8 nm (Fig. 5B). These results supported the confocal microscopy studies and demonstrated that C. jejuni manipulates the nanoscale localization of paxillin in the focal adhesion.

10.1128/mBio.01494-21.3 FIG S3 Representative images of superresolution interferometric photoactivated localization microscopy (iPALM) imaging showing that C. jejuni changes the nanoscale topology of the focal adhesion. (A) Representative iPALM image of a noninfected cell. (B) Representative iPALM image of a C. jejuni-infected cell. (C) Focal adhesions from the noninfected cell shown in panel A. The *Z* position of fluorophores was corrected to the *Z* position of gold fiducials embedded into the coverslip, as described in Materials and Methods in the main text. *Z* position is indicated by color scale gradient. (D) Focal adhesions from the C. jejuni-infected cell shown in panel B. The focal adhesions of C. jejuni-infected cells are displaced upwards compared to noninfected cells. Focal adhesions present in panels A and B but not in panels C and D represent focal adhesions removed from analysis for having an average *Z* position of less than 0 nm. Download FIG S3, TIF file, 2.8 MB. Copyright © 2021 Klappenbach et al. 2021 Klappenbach et al. https://creativecommons.org/licenses/by/4.0/ This content is distributed under the terms of the Creative Commons Attribution 4.0 International license.

### C. jejuni enhances signaling pathways at the focal adhesion.

**FIG 6:**
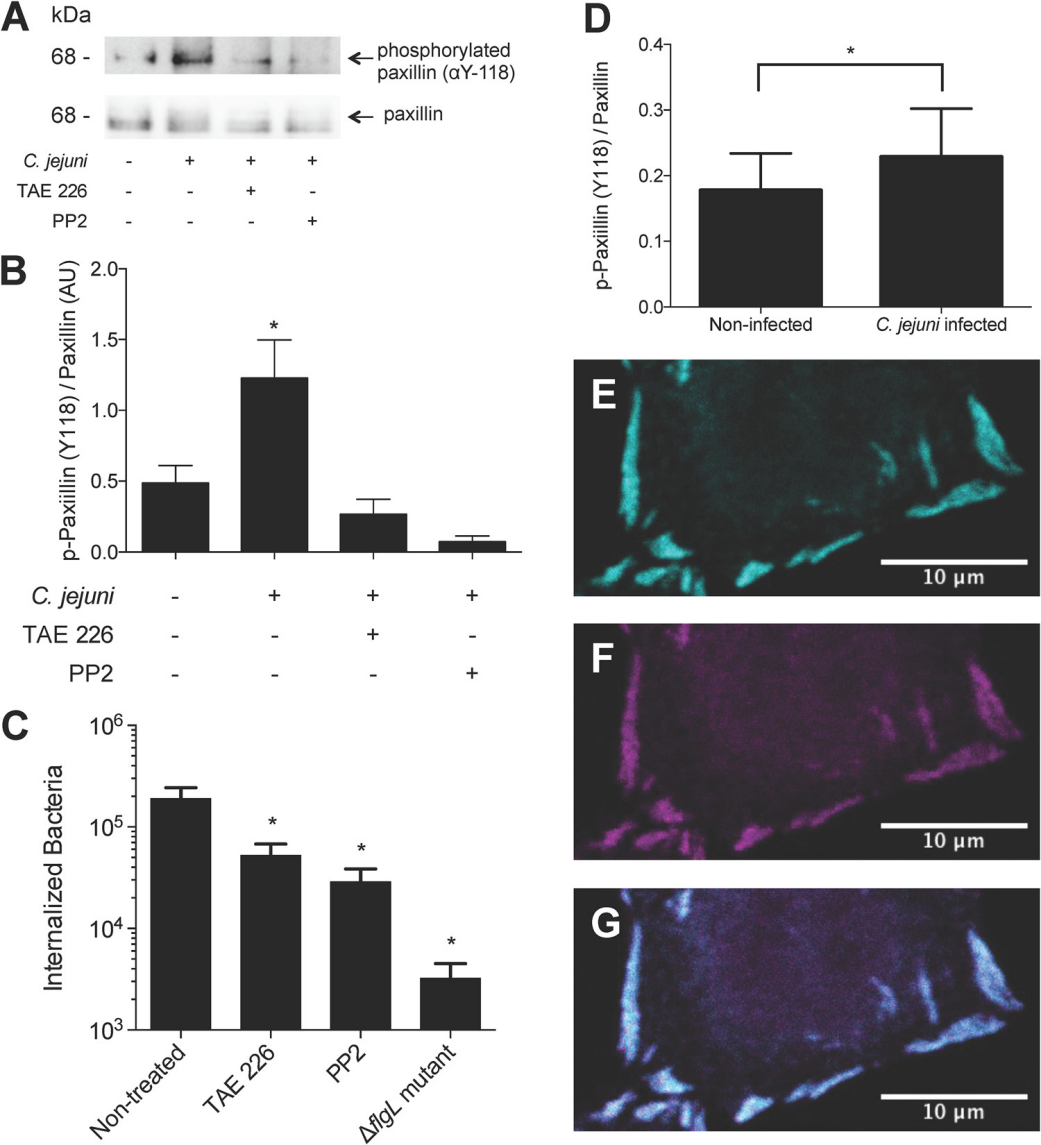
C. jejuni invasion results and paxillin phosphorylation by FAK and Src kinases. (A) INT 407 cells were incubated with C. jejuni for 45 min in the presence of TAE226 (an inhibitor of FAK) or PP2 (an inhibitor of Src). Cells were lysed, and paxillin was immunoprecipitated. SDS-polyacrylamide gels were run, and blots were probed for phosphorylated (Y118) paxillin and total paxillin. (B) Band intensity was measured, and phosphorylated paxillin was normalized by total paxillin. Results showed that C. jejuni causes a significant increase in paxillin phosphorylation but that the addition of TAE226 or PP2 eliminates this effect. Error bars represent standard deviation (SD) between two biological replicates. (C) INT 407 cells were incubated with C. jejuni and inhibitors prior to determining the CFU of internalized bacteria by the gentamicin protection assay. TAE226 and PP2 caused a significant decrease in the number of internalized bacteria compared to that in nontreated cells. Error bars represent standard deviation between technical replicates. *, *P < *0.05 compared to nontreated or noninfected wells (one-way ANOVA with Dunnett's multiple-comparison test). The Δ*flgL* mutant (noninvasive) was used as a negative control. (D to G) INT 407 cells infected with C. jejuni harboring a green fluorescent protein (GFP) plasmid for 60 min show that phosphorylated paxillin is localized to the focal adhesion. Cells were then fixed and stained with a paxillin and phospho-paxillin (Y118) antibody. Focal adhesions were imaged by confocal microscopy. The relative amount of phospho-paxillin was determined by segmenting the focal adhesion and measuring the intensity of paxillin and phospho-paxillin within the segmented area. The phospho-paxillin intensity was divided by the paxillin intensity for each focal adhesion. (D) The relative amount of phospho-paxillin at the focal adhesion increased significantly in C. jejuni-infected cells compared to that in noninfected cells. Error bars represent the standard deviation (SD) of all focal adhesions (>6,000 total sites examined per condition). *, *P < *0.0001 (Student’s *t* test). (E) Representative image of focal adhesions showing paxillin staining only. (F) Focal adhesion showing phospho-paxillin staining only. (G) Composite of images in panels E and F, showing paxillin and phospho-paxillin staining.

Phosphorylation of paxillin (at Tyr118) is indicative of focal adhesion assembly and activation of the focal adhesion kinase (FAK) and Src kinase (47, 48). Previous research has revealed that paxillin is phosphorylated during C. jejuni infection of INT 407 cells in a time-dependent manner (49). To determine if paxillin phosphorylation is related to focal adhesion signaling, we tested the role of FAK and Src kinase in paxillin phosphorylation and C. jejuni invasion. It is well established that these two kinases form a complex together at focal adhesions to phosphorylate many proteins, including paxillin (50). We treated INT 407 epithelial cells with selective FAK (TAE226) and Src (PP2) inhibitors prior to and during infection with C. jejuni. Paxillin was then immunoprecipitated from the cells, and the relative amount of phosphorylated paxillin was determined by immunoblot analysis. Consistent with previous research (49), C. jejuni caused a significant increase in phosphorylated paxillin. Importantly, the presence of the TAE226 or PP2 inhibitors eliminated this effect (Fig. 6A and B). To verify the biological significance of these kinases, C. jejuni invasion in the presence of these inhibitors was determined by gentamicin protection assay. Both kinase inhibitors caused a significant reduction in the number of internalized bacteria (Fig. 6C), which is consistent with previous reports (36, 51–53). To further confirm that the changes in paxillin signaling are related to focal adhesion dynamics, we investigated the phosphorylation of paxillin at the focal adhesion by microscopy. INT 407 epithelial cells were infected with C. jejuni, then fixed and stained with paxillin and phosphorylated paxillin (Y118) antibodies. Cells were imaged by confocal microscopy, and the intensity of paxillin and phosphorylated paxillin was quantified at each focal adhesion. The relative amount of phosphorylated paxillin compared to total paxillin increased significantly in C. jejuni-infected cells compared to that in noninfected cells (Fig. 6D to G). This result demonstrated that the C. jejuni*-*driven paxillin signaling changes are occurring at the focal adhesion.

### Biological significance of focal adhesion manipulation.

**FIG 7:**
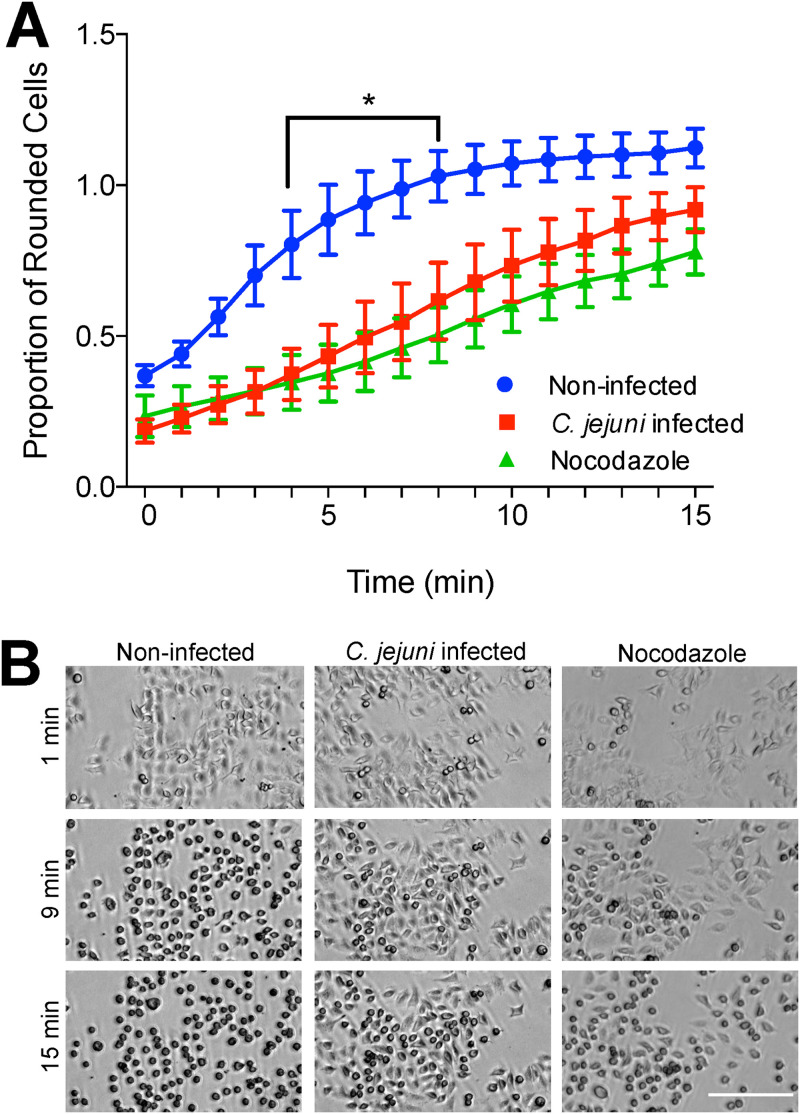
C. jejuni increases host cell adhesion strength. INT 407 cells were seeded 24 h before infection with C. jejuni or treatment with 20 μM nocodazole (a microtubule polymerization inhibitor that increases focal adhesion size and strength) for 45 min. Cells were treated with a low concentration of trypsin to induce cell rounding and then imaged every 5 to 30 s for 15 min. (A) Rounded cells were counted at each minute after trypsin treatment and divided by the total number of cells. The number of rounded cells appearing per minute was larger in noninfected cells than those in C. jejuni-infected or nocodazole-treated cells, indicating increased adhesion strength under these conditions. Error bars represent SEM. *, *P < *0.05 between C. jejuni-infected and noninfected conditions (two-way ANOVA with Sidak’s multiple-comparison test). (B) Images of cells infected with C. jejuni, treated with nocodazole, or noninfected. In noninfected cells, most cells appear rounded at 9 min after trypsin treatment, and all cells are rounded at 15 min. Under nocodazole-treated and C. jejuni-infected conditions, fewer cells are rounded at 9 and 15 min. Bar, 200 μm.

Focal adhesion size has been found to correlate with cellular adherence strength (54, 55). To see if C. jejuni is manipulating focal adhesion strength in addition to size, we used a trypsin-based cell detachment assay. Trypsin is a protease that causes cultured cell detachment. At low concentrations, trypsin treatment can be used to determine adhesion strength (54). Cells that are more strongly attached to the substrate will take longer to detach than cells that are more weakly attached. INT 407 cells were infected with C. jejuni or treated with nocodazole prior to treatment with a low concentration of trypsin. Nocodazole inhibits microtubule polymerization and was used as a positive control, as it increases focal adhesion size and strength (56). Cells were imaged for 15 min with a phase-contrast microscope to observe the cell rounding that precedes detachment. C. jejuni and nocodazole both caused a delay in cell rounding compared to that in noninfected cells (Fig. 7), indicating that they both caused an increase in focal adhesion strength.

**FIG 8:**
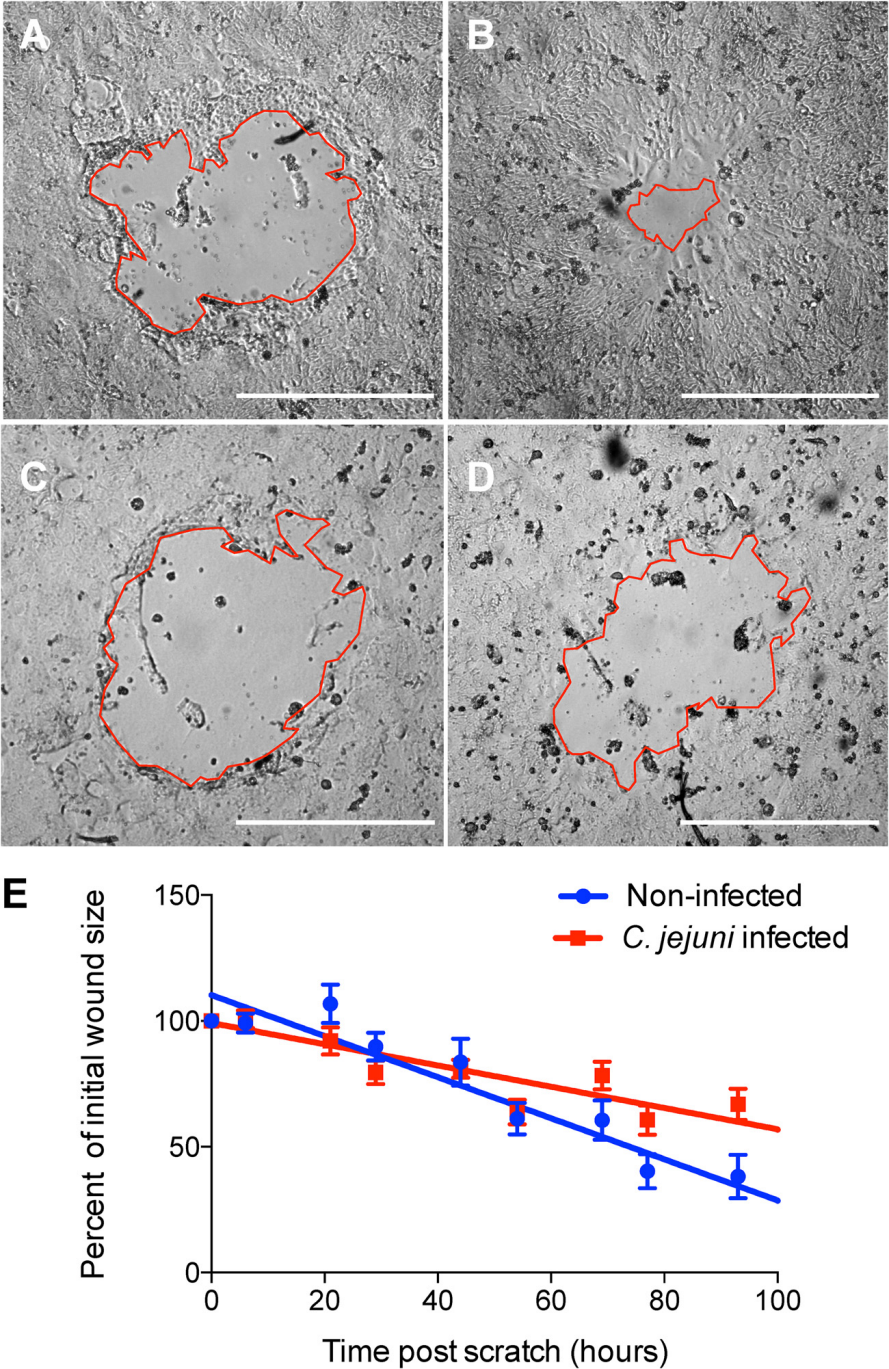
C. jejuni decreases the rate of epithelial wound healing. T84 cells were grown until confluence and then scratched by aspiration to create a small circular wound. Cells were infected with C. jejuni for 3 h and then imaged twice daily for 4 days to monitor wound closure. (A) Noninfected scratch immediately after wounding. (B) Noninfected scratch 100 h after wounding. (C) C. jejuni-infected scratch immediately after wounding. (D) C. jejuni-infected scratch 100 h after wounding. Red lines outline the wound, and bars represent 0.5 mm. (E) Average percentage of initial wound size over time. Lines represent linear regression of points for each condition. The rate of closure is significantly lower for C. jejuni-infected scratches compared to that for noninfected scratches (noninfected = −0.0082 ± 0.00063 and C. jejuni infected = −0.0042 ± 0.00046; *P < *0.0001, analysis of covariance [ANCOVA]).

To understand the effect of focal adhesion manipulation on wound healing, we tested the collective cell migration of human T84 epithelial cells in response to C. jejuni. The human T84 colonic cell line represents an ideal model to mimic tissue-level responses to C. jejuni, as C. jejuni infects the human colon (57, 58). Additionally relevant to these experiments is that T84 cells have been used to mimic the wound healing response in the intestinal villi (15). Small circular scratches were made in an epithelial cell monolayer and imaged for 4 days to observe wound closure. There were no significant differences in cell number or percent viability between infected and noninfected conditions 6 days after scratching cells (data not shown). Noninfected monolayers healed almost completely in 4 days, while monolayers infected with C. jejuni did not (Fig. 8). Linear regression analysis revealed that noninfected scratches healed at a rate of 0.0082 ± 0.00063 (normalized area/hour), while C. jejuni-infected scratches healed significantly slower, at a rate of 0.0042 ± 0.00046 (*P < *0.0001; analysis of covariance [ANCOVA]). The average initial wound size was not significantly different between samples (*P = *0.315; Student’s *t* test). This result shows that C. jejuni significantly decreases the ability of the epithelial cell monolayer to heal wounds.

### Mechanism of C. jejuni focal adhesion manipulation.

**FIG 9:**
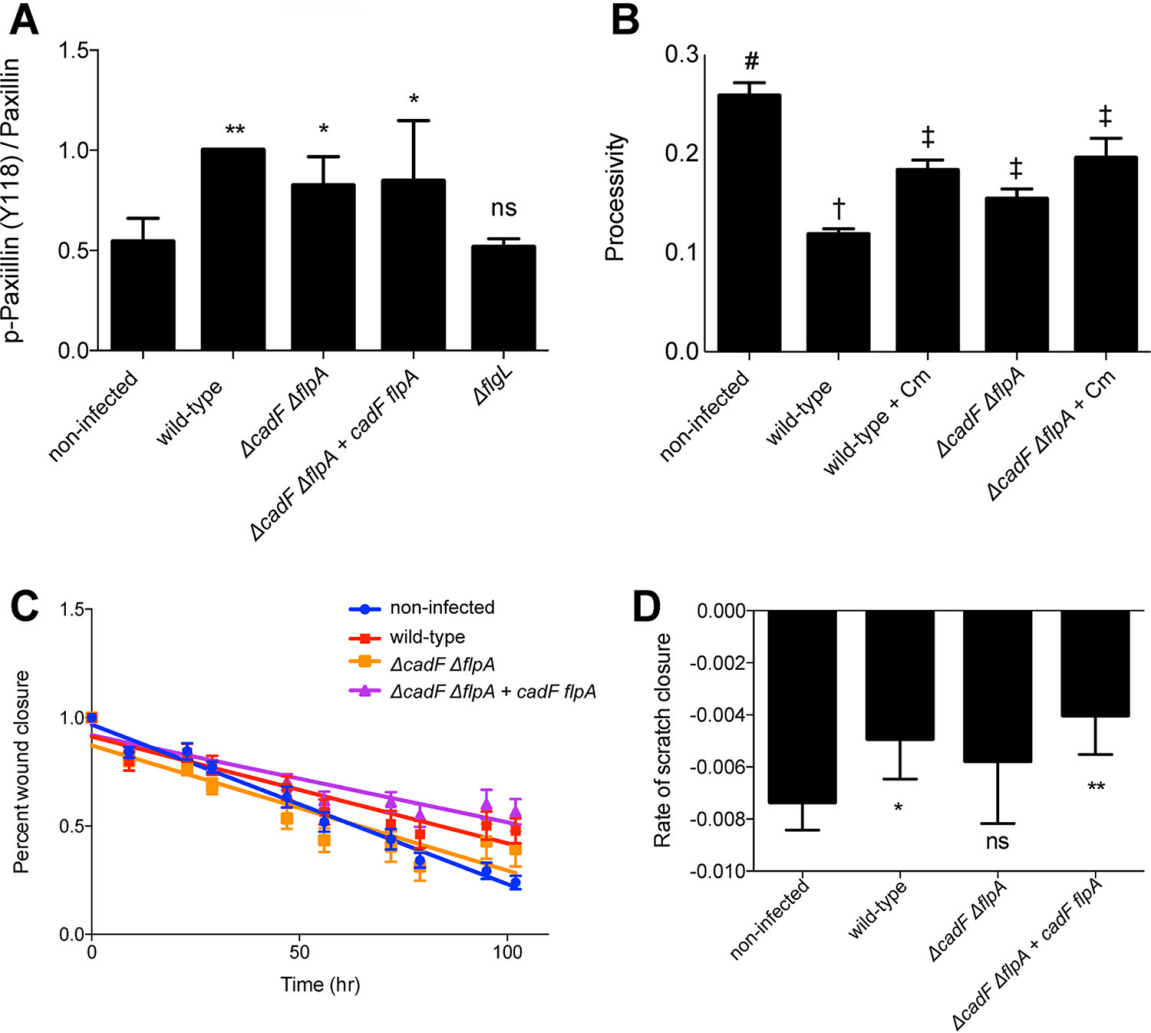
The C. jejuni CadF and FlpA adhesins contribute to focal adhesion modification. (A) INT 407 cells were infected with C. jejuni for 60 min and then lysed. Protein gels were run with whole-cell lysates, and immunoblots were probed for total paxillin and phosphorylated paxillin (Y118). Densitometry was performed to quantify band intensity, and phosphorylated paxillin was normalized to total paxillin. Five biological replicates were normalized to their wild-type condition and averaged. The C. jejuni wild-type caused a significant increase in paxillin phosphorylation compared to that in the noninfected condition. The C. jejuni Δ*flgL* mutant (flagellar hook junction protein; mutant is nonmotile and protein secretion negative) demonstrated low levels of phosphorylation, and the Δ*cadF* Δ*flpA* mutant and complement had a modest reduction in phosphorylation compared to that in cells infected with the C. jejuni wild-type strain. Error bars represent SD. *, *P < *0.5; **, *P < *0.001 by one-way ANOVA with Dunnett’s multiple-comparison test. (B) Migratory A549 cells were grown on a laminin-based extracellular matrix overnight and then infected with the C. jejuni wild-type strain or the C. jejuni Δ*cadF* Δ*flpA* mutant, with and without chloramphenicol to inhibit protein synthesis (and the secretion of the bacterial effector proteins). Cells were imaged for 5 h to observe migration over time. Processivity was calculated to represent directional migration. Wild-type bacteria had significantly decreased processivity; however, migration was partially restored by the addition of chloramphenicol and when the C. jejuni Δ*cadF* Δ*flpA* mutant was used. Use of both the adhesin mutant and chloramphenicol resulted in the highest processivity of all infected conditions. Bars represent the average of >40 cells; error bars represent SEM. By one-way ANOVA with Tukey’s multiple-comparison test: #, significant difference from all other conditions (*P < *0.05); †, significant difference from all other conditions (*P < *0.05); ‡, significant difference from noninfected and wild-type conditions (*P < *0.05). (C) T84 cells were grown to confluence and then scratched by aspiration to create a small circular wound. Cells were left noninfected or were infected with the C. jejuni wild-type strain, the C. jejuni Δ*cadF* Δ*flpA* deletion mutant, or the C. jejuni Δ*cadF* Δ*flpA* + *cadF flpA* complemented isolate. Scratched cells were imaged twice daily for 4 days. Noninfected and C. jejuni Δ*cadF* Δ*flpA* mutant-infected scratches demonstrated a faster closure of the scratch than cells infected with the C. jejuni wild-type or the C. jejuni Δ*cadF* Δ*flpA* + *cadF flpA* complemented strain. 7 to 12 scratches were used per condition. Error bars represent SEM; one of 2 biological replicates. (D) Quantification of the rate of scratch closure for C. jejuni-infected cells. Rate was determined by finding the slope of the line describing normalized scratch area over time for each scratch and averaged. Error bars represent SD. *, *P < *0.01; **, *P < *0.001, compared to the noninfected condition by one-way ANOVA with Dunnett’s multiple-comparison test.

We have shown that C. jejuni manipulates the size, structure, and composition of focal adhesions. Based on previous research connecting C. jejuni adhesins to focal adhesion components (49, 53, 59), we hypothesized that the CadF and FlpA adhesins could be responsible for driving the initial changes in the focal adhesion and secreted effector proteins could contribute to the persistent modulatory effect of the focal adhesion. First, we tested if the CadF and FlpA adhesins were required for signaling changes in the focal adhesion by examining paxillin phosphorylation. INT 407 cells were infected with the C. jejuni wild-type strain, C. jejuni lacking the *cadF* and *flpA* genes (Δ*cadF* Δ*flpA* strain), or the C. jejuni Δ*cadF* Δ*flpA* strain with the *cadF* and *flpA* genes restored (Δ*cadF* Δ*flpA + cadF flpA* strain). Cells were also infected with the C. jejuni Δ*flgL* mutant, as this isolate is deficient in the secretion of the Cia effector proteins. More specifically, the CiaD effector protein, which is not secreted from a flagellar mutant bacterium, activates the MAP kinase signaling pathway, including Erk 1/2, which is in a complex with paxillin (60, 61). Noninfected cells were used as a negative control. We observed a reduced level of phosphorylation in cells infected with the C. jejuni Δ*cadF* Δ*flpA* mutant compared to that in cells infected with the C. jejuni wild-type strain (Fig. 9A; see also Fig. S4). In addition, we observed a reduced level of phosphorylation in cells infected with the C. jejuni Δ*flgL* mutant compared to that in the C. jejuni wild-type strain (Fig. 9A; see also Fig. S4). These findings suggest that the bacterial adhesins and secreted proteins work cooperatively to alter the paxillin activation and downstream signaling pathways.

10.1128/mBio.01494-21.4 FIG S4 Representative blot demonstrating C. jejuni-driven paxillin phosphorylation and dependence on the CadF and FlpA adhesins and the flagellum. Whole-cell lysates from C. jejuni-infected INT 407 cells were analyzed by SDS-PAGE and immunoblotting. Blots were probed for total paxillin and phosphorylated paxillin (Y118). Download FIG S4, TIF file, 0.7 MB. Copyright © 2021 Klappenbach et al. 2021 Klappenbach et al. https://creativecommons.org/licenses/by/4.0/ This content is distributed under the terms of the Creative Commons Attribution 4.0 International license.

To determine how CadF and FlpA may contribute to functional changes to the focal adhesion, we examined their role in individual and collective cell migration. Individual cell migration was tested using A549 epithelial cells. Cells were grown on a laminin-based extracellular matrix, infected with the C. jejuni isolates, and imaged for 5 h to observe migration. In addition to testing the dependence on the adhesins, we wanted to test if metabolically active bacteria were required to limit host cell motility. Therefore, we added chloramphenicol at 256 to 512 μg/ml, a concentration known to prevent the synthesis of the Cia effector proteins (32, 62), resulting in a decrease in bacterial invasion (32). We observed a significant decrease in cell processivity with the C. jejuni wild-type strain (Fig. 9B). Infection of the A549 cells with the C. jejuni wild-type strain with chloramphenicol and infection with the C. jejuni Δ*cadF* Δ*flpA* mutant partially restored processivity. In addition, infection of the A549 cells with the C. jejuni Δ*cadF* Δ*flpA* mutant with chloramphenicol resulted in the highest level of processivity of all infected conditions. In conjunction with the previous finding, these results suggest that the C. jejuni CadF and FlpA fibronectin-binding proteins and secreted effector proteins contribute to C. jejuni focal adhesion modification.

Last, we investigated the dependence of CadF and FlpA in collective cell migration. T84 cells were grown to confluence and then scratched by aspiration to create circular wound beds. Cells were infected with the C. jejuni wild-type strain, the C. jejuni Δ*cadF* Δ*flpA* mutant, or the *cadF*- and *flpA*-complemented isolate. We observed a significantly decreased rate of scratch closure in cells infected with the C. jejuni wild type compared that in to noninfected cells (Fig. 9C and D). Furthermore, we observed that the C. jejuni Δ*cadF* Δ*flpA* mutant had a rate of healing similar to that of the noninfected cells, while the complemented isolate showed the same phenotype as the wild-type condition. Collectively, these results provide evidence that the CadF and FlpA adhesins contribute to the functional and physical changes in the focal adhesion.

## DISCUSSION

The key findings of this study show that C. jejuni changes the structure, composition, and function of cellular focal adhesions using a combination of virulence factors. C. jejuni changes the focal adhesion structure by affecting paxillin footprint size, turnover rate, and nanoscale position. Since paxillin is a dynamic regulator of focal adhesion function, these alterations dramatically change host cell behavior. This was dependent on bacterial virulence factors, including the flagellum and the CadF and FlpA fibronectin-binding adhesins, and required the host cell FAK and Src kinases. The biological outcomes of such manipulations are that cell adhesion, cell motility, and epithelial sheet migration/wound healing were significantly altered by C. jejuni infection. The defects in cell motility are likely due to a disruption in the balanced life cycle of focal adhesions. We propose that the purpose of taking over focal adhesions is driven by C. jejuni needing to utilize structural components for invasion. We have seen these changes by imaging both fixed and live cells, and, to our knowledge, this is the first time C. jejuni has been shown to manipulate the structure and dynamics of the focal adhesion.

An organized balance between focal adhesion assembly and disassembly allows coordinated host cell motility. Research has shown that focal adhesion proteins exist in the following two states: the cytoplasmic pool, which diffuses quickly throughout the cell, and the focal adhesion, which diffuses much more slowly (21). Focal adhesion proteins must assemble from the cytosolic pool to anchor the leading edge of the cell, then disassemble and detach from the trailing edge as the cell moves (47). It is the coordination of these processes that allows cells to have directional migration (20). If focal adhesion turnover is greater than focal adhesion assembly, or if assembly is greater than turnover, cells will be nonmotile (20). We found that C. jejuni increases the size of the focal adhesion, suggesting that assembly may be occurring at a higher rate than turnover. This is further supported by our observation that paxillin localization changes from the cytosol to the focal adhesion during infection. We also observed the focal adhesion to be thicker at the nanoscale level, again pointing to increased protein levels at the focal adhesion. Research has shown that focal adhesion size positively correlates with cell speed until an optimum is reached, at which point it negatively correlates (63). Based on our cell motility results, we hypothesize that during C. jejuni infection, the focal adhesion has increased past this optimum size, resulting in decreased processivity. Focal adhesion assembly and disassembly is a highly regulated process. In addition to the assembly and disassembly balance, individual proteins that compose focal adhesions are constantly joining and leaving the focal adhesion, with each individual component having a unique turnover rate and residency time (21). We investigated the turnover of paxillin at focal adhesions to further understand how assembly was increased during C. jejuni invasion. Interestingly, we found that the turnover of paxillin was decreased in infected cells, suggesting that the increased size of the focal adhesion could be driven by lower turnover (longer residency time) of individual proteins. In support of this model, research has found that larger focal adhesions have an increased protein residency time (21). We hypothesize that C. jejuni increases the focal adhesion footprint size by increasing the residency time of signaling proteins such as paxillin. The most common pathway for paxillin activation is through FAK and Src kinases.

The FAK-Src signaling pathway is required for C. jejuni invasion and paxillin phosphorylation. FAK is a key regulator of focal adhesion dynamics, being implicated in both focal adhesion maturation and turnover (63). Upon integrins binding to fibronectin (21, 50, 64), FAK becomes activated by autophosphorylation on tyrosine 397. This creates a high-affinity SH2 docking site for Src kinase (50, 64, 65). Src then phosphorylates other residues on FAK, and the now-active kinase complex can phosphorylate many focal adhesion proteins, including paxillin (50). Phosphorylated paxillin also has a higher affinity for FAK binding (47), creating a positive feedback loop to recruit more FAK to focal adhesions. Interestingly, previous researchers have found that the phosphorylation of paxillin can influence assembly/disassembly from the focal adhesion (47, 66, 67). We observed that C. jejuni causes the phosphorylation of paxillin at tyrosine 118. Phosphorylation at this site, as well as at tyrosine 31, promotes nascent focal complex formation (10). We also found that C. jejuni infection of cells has decreased paxillin turnover at focal adhesions. These data suggest that C. jejuni utilizes paxillin to manipulate focal adhesions in a unique way, mixing signals for assembly and disassembly to stunt focal adhesion functions (motility, healing, and turnover).

In addition to regulating focal adhesion dynamics, paxillin phosphorylation can also recruit downstream signaling intermediates such as Erk2 and CrkII (10, 50). The phosphorylated tyrosine residues on paxillin create high-affinity SH2 binding sites for the CrkII adaptor protein (35). This results in membrane ruffles via activation of Rac1 by CrkII association with the guanine nucleotide exchange factors (GEFs) DOCK180 and ELMO (47, 68). Rac1 has been shown to be activated during C. jejuni invasion of epithelial cells (41, 69, 70), and DOCK180 is required for maximum invasion (51). We hypothesize that signaling through the focal adhesion leads to membrane ruffling and subsequent invasion. Another role for focal adhesion signaling through paxillin is by acting as a scaffold for Erk. Src-FAK phosphorylation of paxillin at Y118 can lead to Erk association (50, 71), and the MAP kinase cascade has been shown to be activated during C. jejuni infection (61, 72). Activation of host cell signaling has been shown to be dependent on C. jejuni invasion factors, in particular on the CiaD secreted effector; therefore, we investigated what C. jejuni factors were driving changes in the focal adhesion.

We investigated the potential contribution of the C. jejuni adhesins and secreted effectors (via a mutated flagellar apparatus) and observed that both are involved in manipulating the focal adhesion. Regarding the adhesins, it is known that the CadF and FlpA adhesins promote the binding of the bacteria to the fibronectin localized on the basolateral surfaces of cells (73, 74) and stimulate the cell signaling pathways necessary for cell invasion (49, 53, 73). We also found that a functional flagellum/secretion apparatus was required for C. jejuni to increase the size of the focal adhesion and limit host cell motility using Δ*flgL*, Δ*flhF*, and Δ*cheB* mutants (32, 42). We propose a model whereby the CadF and FlpA adhesins promote host cell contact, which permits the delivery of effector proteins and further modification of host cell signaling pathways. This proposal is consistent with the finding that the combination of adhesin mutant and the use of chloramphenicol led to the greatest individual cell motility of all infected conditions. Further research is required to define the precise role of the adhesins and secreted proteins in the modification of the focal adhesion and their impact on individual and collective cell migration.

It is common for bacterial pathogens to target the focal adhesion, due to the nature of focal adhesions in signaling between the extracellular matrix and the cytoskeleton. Salmonella Typhimurium is an extensively studied intestinal pathogen that invades host cells by secreting effector proteins into the cell. Several focal adhesion proteins have been implicated in Salmonella invasion. Shi et al. found that Salmonella recruits FAK, Cas, and paxillin to sites of bacterial invasion, creating focal-adhesion-like structures. Interestingly, these structures form on the apical rather than the basal cell surface of polarized cells and do not include beta integrins (31). This is in contrast to C. jejuni, where we observed an increase in the size of preexisting focal adhesions rather than the creation of new focal adhesion-like structures surrounding the bacterium. Furthermore, Shi et al. found that FAK and Cas are required for bacterial invasion but that the catalytic domains of FAK are not (31). In agreement with this observation, inhibition of Src kinase by PP2 did not decrease invasion (31). This is again in contrast to the results we observed with C. jejuni, where the catalytic activities of Src and FAK are required for invasion and paxillin phosphorylation. Previous research has also shown that talin and α-actinin, structural components of the focal adhesion, assemble at sites of Salmonella bacterial invasion (75). It is still unknown what effectors are responsible for the recruitment of FAK and Cas (76) and how the pathway connects to invasion. However, a recent study has shown that in macrophages, SPI-2 effector proteins target FAK to avoid fusion of the Salmonella containing vacuole with the lysosome (77). In addition, *S.* Typhimurium infection of bone marrow-derived macrophages causes irregular movement and decreased directional movement. This is dependent on Sse1, an effector of SPI-2 (78). This result is consistent with our finding that Salmonella decreased the processivity of A549 epithelial cells. While macrophages utilize podosomes rather than focal adhesions for motility, many of the regulatory proteins, such as FAK and paxillin, are involved in both (79).

We observed by the trypsin detachment assay that C. jejuni increases focal adhesion attachment strength. The epithelial cells of intestinal villi are regularly shed by cell extrusion and programmed cell death at the villus tips. New cells migrating up from the crypts then replace these lost cells. An equilibrium between division and migration from the crypts and shedding at the tips keeps the number of cells constant (80). Pathogens take advantage of this natural process to cause disease. Listeria monocytogenes invade the villi at sites of extrusion, taking advantage of the briefly available E-cadherin (81). Other enteric pathogens block extrusion to prevent infected cells from being shed (82). The OspE effector, which is conserved in Shigella flexneri, enteropathogenic Escherichia coli (EPEC), enterohemorrhagic E. coli (EHEC), Salmonella, and Citrobacter rodentium, interacts with integrin-linked kinase (ILK) to block epithelial cell turnover and increase cell adhesion to the matrix (82, 83). In addition, this interaction decreases wound healing, blocks focal adhesion turnover, and increases the number of focal adhesions (82, 83). This is consistent with our observations that C. jejuni increases adhesion strength, decreases turnover, and blocks migration. However, OspE interaction with ILK causes decreases in paxillin and FAK phosphorylation, suggesting that the pathway by which C. jejuni causes these changes differs from the OspE pathway. Pathogens often increase host focal adhesion strength to prevent cell extrusion from the villi. This manipulation can be twofold, as altering focal adhesion dynamics can also prevent the villi from healing.

The piglet model of C. jejuni disease has shown that infection results in cell necrosis and villus blunting (26). The process of repairing damage is termed restitution (14–18). Most research has been done on wounds created by ischemia, viral infection, parasites, or chemicals (18). However, villus blunting and denuding as the epithelium lifts from the basement membrane is common in all of these agents (14, 17, 18, 84). Several sequential steps are involved in repairing villus damage. First, the villus contracts to shed damaged cells (14, 85). Next, viable cells depolarize and flatten, extending lamellipodial membrane protrusions into the damaged area (14–18). These cells then migrate to cover the damaged area. This process is dependent upon focal adhesions assembling on the leading edge of the cell and disassembling to detach from the trailing end, similarly to single-cell motility (14). Finally, tight junctions are restored as the villus wound closes (14, 16). For *in vitro* studies, T84 cells are commonly used (15, 85, 86). We observed that C. jejuni decreases the collective migration of T84 cells (wound healing) and hypothesize that this is due to focal adhesion manipulation. In support of this hypothesis, this effect has been observed in E. coli. The cytotoxic necrotizing factor 1 (CNF1) from E. coli has been shown to decrease T84 wound healing (86). CNF1 has also been shown to cause paxillin and FAK phosphorylation, further supporting the idea that migration is blocked due to manipulation of focal adhesion components (86).

We have laid the groundwork for understanding how C. jejuni manipulates the focal adhesion during infection by performing a mechanistic study focusing on the functions of focal adhesions. We observed that the focal adhesion size, *Z* position, and thickness increase in response to infection. We observed that these phenotypes were driven by well-known C. jejuni virulence determinants that, together, allow the changes in focal adhesion structure and signaling function. We hypothesize that these events have biological significance in prolonging C. jejuni infection of the human intestine. To summarize, we have identified a new cellular phenotype in C. jejuni-infected cells subsequent to cellular invasion. Studies are ongoing to understand how focal adhesion manipulation is directed toward early infection signaling to allow invasion and late-infection manipulation to prolong sickness in a host.

## MATERIALS AND METHODS

### Bacterial strains and growth conditions.

Campylobacter jejuni strain 81-176 was cultured on Mueller-Hinton agar (Hardy Diagnostics, Santa Maria, CA) containing 5% citrated bovine blood (MHB agar) under microaerobic (10.5% CO_2_) conditions at 37°C in a Napco 8000WJ incubator (Thermo Fisher, Waltham, MA). The bacteria were subcultured on MHB agar every 24 to 48 h. Before infection of cultured epithelial cells, C. jejuni were grown overnight on an MHB plate overlaid with MH broth (biphasic conditions). Salmonella enterica serovar Typhimurium (*S.* Typhimurium) SL1344 and Staphylococcus aureus ATCC 25923 were cultured on LB agar and LB broth as needed. Prior to use, the Salmonella culture was diluted 1:80 and grown for 2.5 h to reach the log phase, which corresponded to an optical density at 600 nm (OD_600_) of ∼0.8 (87).

### Generation and culture of C. jejuni deletion mutants and complemented isolates.

The C. jejuni 81-176 Δ*flgL*, Δ*cheB*, and Δ*flhF* mutants and the *flgL*-complemented isolate were generated as outlined elsewhere (32). The C. jejuni 81-176 Δ*cadF* Δ*flpA* mutant and complemented isolate were generated as described elsewhere (73). The Δ*flgL*, Δ*flhF*, and Δ*cheB* mutant were passaged on MHB agar plates supplemented with 8 μg/ml of chloramphenicol, and the *flgL*-complemented isolate, Δ*cadF* Δ*flpA* mutant, and *cadF*- and *flpA*-complemented isolate were passaged on MHB agar plates supplemented with 250 μg/ml of hygromycin B.

### Cell lines.

Epithelial INT 407 (ATCC CCL-6), epithelial lung carcinoma A549 (ATCC CCL-185), epithelial colonic carcinoma T84 (ATCC CCL-248), and rat bladder carcinoma cell line 804G were cultured in minimal essential medium (MEM; Gibco, Grand Island, NY) supplemented with 10% fetal bovine serum (FBS; heat-inactivated Seradigm premium-grade fetal bovine serum; VWR, Radnor, PA) and 1 mM sodium pyruvate (Corning Inc., Manassas, VA) at 37°C with 5% CO_2_. The A549 and 804G cells were a kind gift of Jonathan C. R. Jones (WSU, Pullman, WA).

### Immunofluorescence microscopy.

INT 407 cells were seeded onto glass coverslips in a 24- or 6-well dish approximately 24 h before infection. Cells were seeded at 3 × 10^4^ cells/well for 24-well dishes or at 1.5 × 10^5^ cells/well for 6-well dishes. C. jejuni was collected from biphasic conditions and adjusted to an OD_540_ of 0.1 in MEM supplemented with 1% FBS. Cells were rinsed once with 1% FBS-MEM before adding C. jejuni and then incubated at 37°C in 5% CO_2_ for 60 min unless otherwise indicated. Following incubation, cells were fixed with 4% paraformaldehyde for 5 to 10 min. Cells were then rinsed and permeabilized with 0.1% Triton X-100 in phosphate-buffered saline (PBS) containing 0.3% bovine serum albumin. C. jejuni was stained using a polyclonal rabbit anti-C. jejuni antibody (1:4,000), paxillin was stained with a mouse anti-paxillin antibody (1:250; BD Biosciences, San Jose, CA), and phospho-paxillin was stained with a rabbit anti-phospho-paxillin antibody (Y118, 1:50; Cell Signaling Technologies). Secondary antibodies that were used for visualization included an Alexa Fluor 680-conjugated anti-rabbit antibody (1:1,000, Jackson ImmunoResearch, West Grove, PA) and an Alexa Fluor 594-conjugated anti-mouse antibody (1:1,000, Jackson ImmunoResearch). Where necessary, actin was stained with fluorescein isothiocyanate (FITC)-conjugated phalloidin (1:1,000, Sigma-Aldrich, St. Louis, MO). Images of cells infected with C. jejuni were taken with a confocal microscope (TCS SP5 and a TCS SP8 with Lightning; Leica Microsystems). Images were quantified with Fiji. To measure focal adhesion size, the paxillin channel was used. The background was subtracted, and a threshold was set that included only focal adhesions greater than 0.25 μm^2^. Focal adhesions from all images were combined to ensure that >1,000 focal adhesions were analyzed per condition. Focal adhesions more than 7 standard deviations away from the mean were removed from subsequent analysis. For quantification of paxillin localization, the C. jejuni channel was removed, and images were randomized. Images were scored in a blind manner based on paxillin localization (0 = paxillin at the focal adhesion, 2 = paxillin in the cytosol) by a trained individual who did not help in image acquisition. For phospho-paxillin, the focal adhesion location was measured as described above, and then all focal adhesions were added to the region of interest (ROI) manager. The intensities (mean gray value) of the paxillin and phospho-paxillin channels at the focal adhesions were measured from the ROI manager. Then, the phospho-paxillin intensity was divided by the paxillin intensity at each focal adhesion.

### iPALM microscopy.

iPALM images were captured using a similar procedure to one described previously (46). Briefly, INT 407 cells were seeded on a gold-fiducial-coated coverslip and grown overnight prior to transfection with a tdEos–paxillin-expressing plasmid using Lipofectamine 3000. Approximately 18 h after transfection, the cells were infected with C. jejuni bacteria that were collected from a biphasic culture and adjusted to an OD_540_ of 0.3. The cells were infected for 45 min prior to fixation in 0.4% paraformaldehyde and 0.1% glutaraldehyde in PBS for 5 min. Samples were washed with PBS and quenched with 1% NaBH_4_. Immunostaining for C. jejuni was performed as described above. Images were taken with a laser power intensity of 2 kW/cm^2^ and an exposure time of 50 ms. The number of frames acquired per image was 25,000. PeakSelector software was used to localize data, perform drift correction, and render images.

### iPALM data processing.

ImageJ was used to process the image output files. Focal adhesions were selected from two-dimensional tiff outputs of iPALM images by smoothing the image (Gaussian blur), thresholding, and analyzing particles larger than 0.5 μm (2) in order to generate regions of interest (ROI). The ROI data were used to process the data output from iPALM imaging in R Studio. A locally estimated scatterplot smoothing (LOESS) regression was performed on the gold fiducials. All fluorophores were normalized to their nearest neighbor of the LOESS regression. The *Z* positions of all fluorophores within a focal adhesion, as determined by each ROI, were averaged. Focal adhesions with *Z* positions calculated as less than 0 nm were removed from further analysis. Then, fluorophores with *Z* positions of less than 0 nm were removed. The average Z position of all focal adhesions was determined and compared between noninfected and C. jejuni-infected cells. The thickness of the focal adhesion was determined as the interquartile range (75th percentile minus 25th percentile) of all fluorophores within a focal adhesion.

### Live cell imaging to determine cell adhesion strength.

INT 407 cells were seeded onto 35-mm tissue culture petri dishes at 4.5 × 10^5^ cells/dish approximately 24 h before infection, with three dishes per condition. For some experiments, cells were seeded in a 24-well dish at 9.4 × 10^4^ cells/well, and eight wells were used per condition. C. jejuni from a biphasic culture was adjusted to an OD_540_ of 0.375 in 1% FBS-MEM. Cells were rinsed once with 1% FBS-MEM before adding the C. jejuni. For nocodazole-treated cells, 20 nM nocodazole in 1% FBS-MEM was added to cells after rinsing. For noninfected cells, 1% FBS-MEM alone was added. Cells were incubated for 45 min at 37°C before imaging. Cells were rinsed once with PBS, and then a low concentration (0.05%) of trypsin with 684.4 μM EDTA was added to induce cell rounding. A phase-contrast microscope (Eclipse TE2000-U; Nikon) was used to capture an image every 5 to 30 s. Images were collected for 16 min. At each minute, rounded cells were identified by the Trainable Weka Segmentation Fiji plugin or by Ilastik trainable image segmentation software trained to identify rounded cells. The number of rounded cells at each minute was divided by the total number of cells counted in the first image.

### A549 motility.

To collect the extracellular matrix, 804G cells were grown to confluence and incubated for 6 days. The supernatant (conditioned medium) was collected, spun, filtered, and frozen until use. Twelve-well dishes were coated with the 804G conditioned medium for 2 to 4 h, then rinsed with PBS before seeding A549 cells at a density of 7.5 × 10^3^ cells/well at 16 to 20 h before infection. Experiments were conducted on an automated Leica DMi8 microscope with a heated stage. C. jejuni bacteria were collected from a biphasic culture, and the OD_540_ was adjusted to 0.1 in 10% FBS-MEM. For some experiments, the 10% FBS-MEM was made with reduced sodium bicarbonate (1.2 mM) and 10 mM HEPES. For some experiments with the C. jejuni Δ*cadF* Δ*flpA* mutant and the *cadF*- and *flpA*-complemented isolate, cells were preincubated with 1 ml of 0.1% FBS-MEM for 30 min, and then 0.5 ml of 30% FBS MEM was added immediately prior to imaging to bring the total concentration of FBS to 10%. For experiments with chloramphenicol (GoldBio, St. Louis, MO), the bacteria were incubated with the drug for 30 min prior to cell infection to reduce invasion but not bacterial viability (256 μg/ml or 512 μg/ml [32, 88]). Chloramphenicol was maintained for the duration of the infection and imaging. Cells were imaged for 5 h, with one image taken every 5 min. Images were processed with an in-house localization software and the TrackMate Fiji plugin. All code used to process the images is available as open-source software at https://github.com/nimne/ACIT. Statistical analysis and wind rose plots were done using R. Processivity was calculated as path distance divided by total displacement every 5 min and averaged for all cells. For experiments using *S.* Typhimurium and S. aureus, A549 cells were preincubated with bacteria in 10% FBS-MEM with 26.2 mM sodium bicarbonate for 60 min in a 5.5% CO_2_ 37°C incubator. Cells were then rinsed 3 times with PBS to remove excess bacteria, then imaged as described above in low sodium bicarbonate 10% FBS-MEM. Cells were infected with approximately 2.4 × 10^8^ CFU of *S.* Typhimurium and S. aureus. Chloramphenicol was added to prevent bacterial replication during the assay (8 μg/ml for *S.* Typhimurium and 64 μg/ml for S. aureus).

### Paxillin immunoprecipitation for inhibitor experiments.

INT 407 cells were seeded at a density of 6 × 10^5^ cells/well in 6-well tissue culture trays and incubated at 37°C in a humidified 5% CO_2_ incubator overnight. Cells were rinsed with MEM lacking FBS and infected with the C. jejuni wild-type strain and the Δ*flgL* mutant at an OD_540_ of 0.3 and incubated for 45 min. After the incubation, the cells were rinsed three times with ice-cold PBS and lysed by the addition of ice-cold immunoprecipitation (IP) lysis buffer (25 mM Tris-HCl [pH 7.5], 1 mM EDTA, 50 mM NaF, 150 mM NaCl, 5% glycerol, 1% Triton X-100, 1× protease inhibitor cocktail [Sigma], 1 mM Na_3_VO_4_, and 1× Halt phosphatase inhibitor cocktail [Thermo Scientific, USA]) and incubated for 20 min on ice. The lysate was clarified by centrifugation at 14,000 rpm for 15 min at 4°C. Immunoprecipitation was performed by incubating mouse anti-paxillin antibody (1:250; BD Biosciences, USA) with the lysate overnight at 4°C, followed by addition of protein A/G Plus-agarose beads (Santa Cruz Biotechnology, Dallas, TX) and incubation for 2 h at 4°C. The precipitate was rinsed four times with ice-cold IP wash buffer (20 mM HEPES, 150 mM NaCl, 50 mM NaF, 1 mM Na_3_VO_4_, 0.1% Triton X-100, 10% glycerol, 1× protease inhibitor cocktail, and 1× phosphatase inhibitor cocktail). Samples were analyzed by SDS-PAGE and immunoblot analysis, as outlined previously (49). Immunoblot detection of phosphorylated paxillin was performed using a 1:2,000 dilution of a mouse anti-phospho-paxillin antibody (Y-118; Cell Signaling Technology, Danvers, MA). Detection of the total pool of paxillin was performed using a 1:1,000 dilution of a mouse anti-paxillin antibody and a 1:4,000 dilution of a peroxidase-conjugated rabbit anti-mouse IgG.

### Paxillin phosphorylation for *cadF flpA* deletion mutant experiments.

INT 407 cells were seeded at 3.6 × 10^6^ cells/well in 100-cm^2^ dishes or 9.2 × 10^4^ cells/well in 24-well dishes at 20 to 28 h prior to infection. Cells were rinsed with MEM lacking FBS one time and then incubated with MEM lacking FBS for 3 to 4 h. C. jejuni bacteria were collected from a biphasic culture, and the OD_540_ was adjusted to 0.035. The C. jejuni suspension was added in a volume equal to that of the volume in the well used for the serum starvation. After 60 min of infection, cells were treated with a lysis buffer (25 mM Tris HCl, 150 mM NaCl, 5% glycerol, 1% Triton X-100, 1× protease inhibitor cocktail [Thermo Scientific], and 2× phosphatase inhibitor cocktail [Thermo Scientific]) on ice for 10 min before collection of cell lysates by scraping. For experiments in 24-well dishes, cells were lysed with 2× sample buffer on ice for 5 min before collection. Lysate samples were analyzed by SDS-PAGE and immunoblot analysis, as outlined previously (49). Immunoblot detection of phosphorylated paxillin was performed using a 1:1,000 dilution of a mouse anti-phospho-paxillin antibody (Y-118; Cell Signaling Technology, Danvers, MA). Detection of the total paxillin was performed using a 1:1,000 dilution of a mouse anti-paxillin antibody. A 1:4,000 dilution of a peroxidase-conjugated rabbit anti-mouse IgG was used to detect both primary antibodies.

### Binding and internalization assays.

C. jejuni binding and internalization assays were performed with INT 407 cells, as outlined elsewhere (88). All assays were performed at a multiplicity of infection (MOI) that ranged between 50 and 500 and repeated a minimum of 3 times to ensure reproducibility. The reported values represent the mean counts plus or minus standard deviations derived from quadruplicate wells. To test the effect of FAK and Src inhibition on C. jejuni cell invasion, INT 407 cells were preincubated for 30 min in MEM containing 5 μM TAE 226 (Selleck Chemicals, Houston, TX) or 10 μg/ml PP2 (Sigma) in 0.5 ml of medium. Following incubation, a 0.5-ml suspension of C. jejuni in MEM was added to each well, and the binding and internalization assays were performed using standard laboratory protocols. To determine if a drug affected the viability of the INT 407 cells, the cells were rinsed twice with PBS following inhibitor treatment, stained with 0.5% trypan blue for 5 min, and visualized with an inverted microscope.

### Measurement of paxillin turnover.

INT 407 cells were seeded at 1.5 × 10^5^ cells/well in glass-bottomed tissue culture dishes. The next day, cells were transfected with a tdEos–paxillin-expressing plasmid using Lipofectamine 3000 transfection reagents and incubated for 16 to 20 h. C. jejuni was collected from a biphasic culture, and the OD_540_ was measured. C. jejuni was diluted in 10% FBS-MEM with 1.2 mM sodium bicarbonate and 10 mM HEPES to an OD_540_ of 0.25. Cells were incubated with C. jejuni or medium alone for approximately 60 min before imaging. Cells were imaged with a point-scanning confocal microscope with a heated stage (TCS SP8X; Leica Microsystems). Individual cells were selected, and half of each cell was photoswitched with a 405 nm laser. Each cell was imaged every 5 s for 300 s after photoswitching. Multiple cells were imaged to ensure that more than 50 focal adhesions were examined. Images were analyzed with Fiji. At each focal adhesion of >0.25 μm^2^, the intensity of red fluorescence was measured in every frame. The slope of the red fluorescence in the nonphotoswitched half of the cell was calculated by linear regression analysis.

### Collective cell migration.

T84 cells were seeded at 2.25 × 10^5^ cells well in 24-well tissue culture dishes. Cells were given fresh 10% FBS-MEM every day until confluence was reached (7 to 12 days). Cells were scratched by aspiration with a gel-loading or 0.1- to 2-μl tip. Immediately following scratching, cells were infected with C. jejuni. C. jejuni bacteria from a biphasic culture were adjusted to an OD_540_ of 0.3 in 1% FBS-MEM and added to cells for 3 h. After infection, the medium was replaced with 1% FBS-MEM. Scratches were imaged with a phase-contrast microscope 1 or 2 times daily, and the medium was replaced every day for 4 days to monitor wound healing. 1% FBS MEM was used to prevent cell replication into the scratches. For each condition, 6 to 12 scratches were averaged. The reported values for linear regression slopes represent the mean counts ± standard error of the mean (SEM).

### Statistical analysis.

All statistical analysis was done in Prism v6.0a (GraphPad Software, Inc.) or R v3.5.1. Biological replicates were defined as experiments repeated on separate days, and one of three replicates is shown for each figure unless otherwise noted.
