# Supplementary figures and images for: Campylobacter jejuni Triggers Signaling through Host Cell Focal Adhesions To Inhibit Cell Motility
Source: mBio. 2021 Aug 24;12(4):e01494-21. doi: 10.1128/mBio.01494-21 (PMC8406305; doi:10.1128/mBio.01494-21)

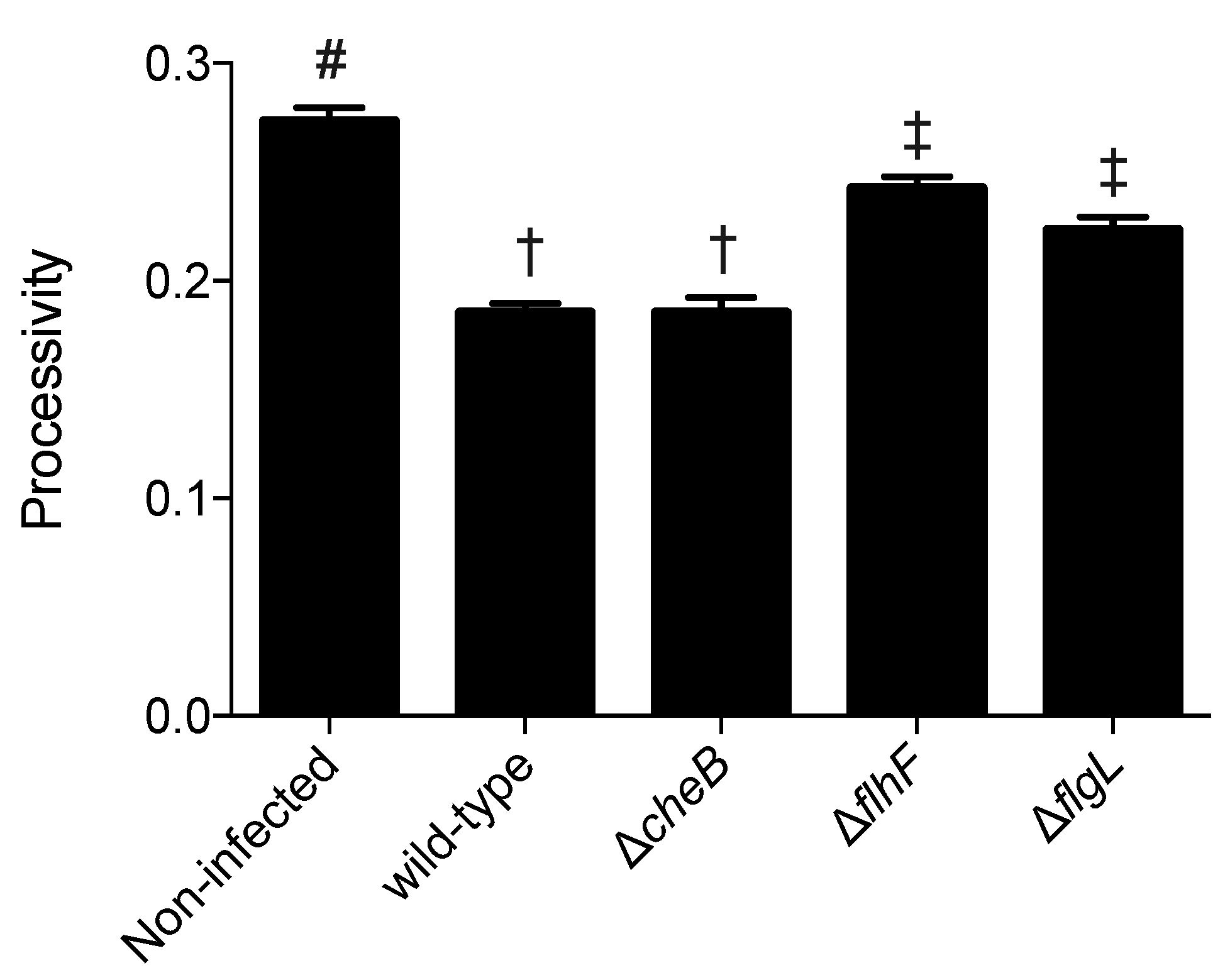

Supplement: FIG S2 [file mbio.01494-21-sf002.tif]

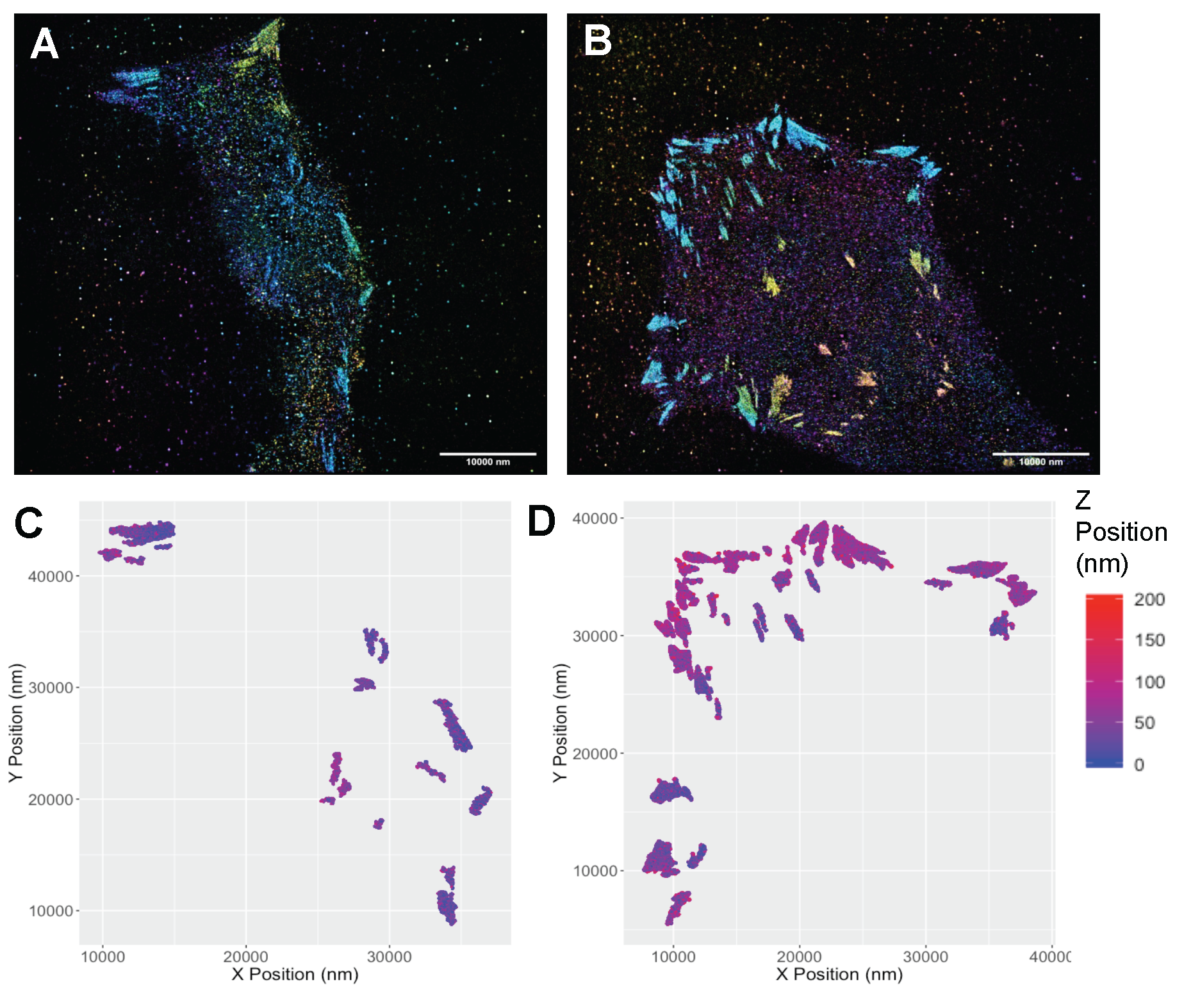

Supplement: FIG S3 [file mbio.01494-21-sf003.tif]

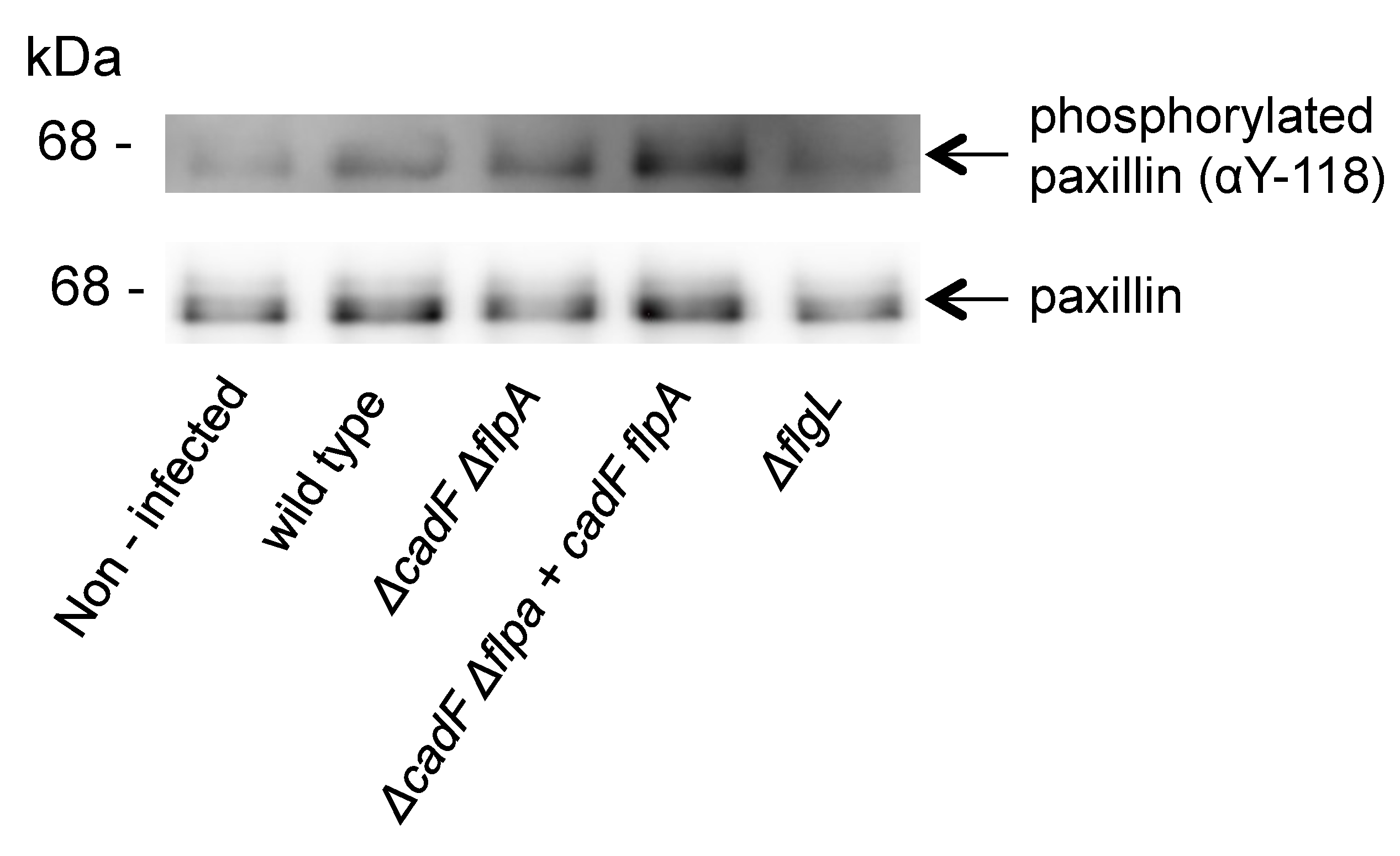

Supplement: FIG S4 [file mbio.01494-21-sf004.tif]
